# Response of soil bacterial community structure in Pisha sandstone areas to different stumping

**DOI:** 10.7717/peerj.21234

**Published:** 2026-05-04

**Authors:** Lu Cheng, Guihua Wang, Yuefeng Guo, Zhiqiang Zhang, Chengfu Zhang, Min Han, Yajie Feng, Yang Song

**Affiliations:** 1 College of Desert Control Science and Engineering, Inner Mongolia Agricultural University, Hohhot, Inner Mongolia, China; 2 Old Barag County Forestry and Grassland Bureau Comprehensive Support Center, Hulun Buir, Inner Mongolia, China

**Keywords:** Pisha sandstone areas, *Hippophae rhamnoides*, Illumina MiSeq, Bacterial community diversity

## Abstract

**Background:**

The Pisha sandstone region of Inner Mongolia is one of the areas most severely affected by soil erosion in the world. Stumping is a widely used practice for rejuvenating degraded sea buckthorn (*Hippophae rhamnoides*) plantations, but how stumping depth affects soil bacterial communities and the underlying drivers remains unclear. We hypothesized that (1) 15 cm stumping depth would maximize soil nutrient availability and bacterial diversity, and (2) shifts in bacterial community composition would be primarily driven by soil nutrients and physical properties.

**Methods:**

We conducted a field experiment in a 10-year-old declining sea buckthorn plantation, applying four stumping heights (0 cm, 10 cm, 15 cm, 20 cm) with uncut forest as control. Soil samples were collected from three compartments (root surface, rhizosphere, non-rhizosphere). Bacterial community composition was characterized by high-throughput sequencing of the 16S rRNA gene V4 region using primers 515F/806R with high-fidelity DNA polymerase. Soil physicochemical properties were measured using standard methods.

**Results:**

Stumping at 15 cm height consistently exhibited the strongest effects across all soil compartments. Compared to other treatments and the control, this treatment significantly increased soil organic carbon, available nitrogen, phosphorus, and potassium, and enhanced bacterial α-diversity (Chao1, Shannon). At the family level, 15 cm stumping most strongly enriched * Oxalobacteraceae*, with relative abundance peaking at 38.9% in rhizosphere soil. Bacterial community composition shifted with stumping depth; however, β-diversity variation was primarily explained by spatial heterogeneity among the three plots (*R*^2^ = 11.89%, *P* = 0.008), rather than stumping treatment itself (*R*^2^ = 9.89%, *P* = 0.226). Redundancy analysis further revealed that *Actinobacteriota* abundance was positively correlated with soil organic matter, available nitrogen, available phosphorus, available potassium, and soil water content, but negatively correlated with soil bulk density. Membership function analysis confirmed that 15 cm stumping yielded the highest comprehensive scores across all soil compartments, particularly in root surface soil (0.54, 0.53, and 0.52 for root surface, rhizosphere, and non-rhizosphere soils, respectively). Taken together, these findings demonstrate that 15 cm stumping effectively improves soil nutrient conditions, enriches beneficial bacterial taxa, and reshapes microbial communities in degraded sea buckthorn plantations, with the strongest effects occurring in root-associated soils.

## Introduction

The Pisha sandstone area of Inner Mongolia is one of the regions most severely affected by soil erosion in the world (Ambuque, 2013). Due to the unique properties of Pisha sandstone, which turns into mud when exposed to water and sand when exposed to wind, the ecological environment of this region is fragile. The area is a major source of coarse sand for the Yellow River’s sediment (Yuefeng et al., 2021), which not only leads to significant sediment deposition in the river’s lower reaches but also triggers a series of serious ecological and environmental issues (Lijiu, Changming & Jingliang, 2013; Qi-Xin et al., 2019; Pan et al., 2022). Sea buckthorn (*Hippophae rhamnoides*) is a shrub or small tree characterized by a well-developed root system, strong tillering ability, and rapid reproduction. In the Pisha sandstone area of Inner Mongolia, sea buckthorn is valued for its unique ecological benefits (Yuefeng et al., 2020). Additionally, sea buckthorn demonstrates strong soil adaptability and environmental resilience (Zhenqi et al., 2019; Lina et al., 2015). However, in recent years, environmental factors such as scarce rainfall and the region’s arid climate have caused the decline or even death of large areas of sea buckthorn forests. The practice of stumping—cutting back the above-ground branches of shrubs or semi-shrubs to stimulate new growth from the rootstock—has been shown to significantly promote the growth of suckers in the above-ground parts of sea buckthorn stands and enhance the vitality of the root system (Yuefeng et al., 2020). Soil harbors microbial communities with a high degree of diversity, which may contribute to plant growth, nutrient uptake, disease resistance, and stress tolerancey (Maestre et al., 2015). The microbiota in soil (non-inter-root, inter-root, and root surface) is directly or indirectly influenced by biotic and abiotic factors, such as climatic conditions and soil physico-chemical properties (Yangquanwei et al., 2022). Plants and microorganisms in the inter-root soil engage in reciprocal feedforward regulation and are closely linked organisms (Shijun et al., 2012).

The development of modern molecular biotechnology is advancing rapidly, and many researchers—both domestically and internationally—are focusing on the study of soil microorganisms, which have become an important indicator for evaluating ecological environment quality (Li et al., 2016). Recent studies indicate that different reclamation methods significantly affect soil microbial populations (Yang, 2020; Wenqin et al., 2008). Investigations into soil microbial communities in reclaimed mining areas across diverse regions have revealed both positive and negative correlations between soil salinity and microbial community structure and abundance  (Jianjun, 2007; Yuanzheng, 2009; Baldwin et al., 2006). Researchers have used the BIOLOG-ECO microplate assay and dilution plate method to assess soil microbial community structure (Chao et al., 2021; Yuxin et al., 2010; Zhao et al., 2016). However, due to methodological limitations, these studies cannot fully reflect the structural and quantitative characteristics of soil microbial communities (Aimei et al., 2018; Mingming, 2019). Currently, fewer studies have examined the response of soil microbial (non-rhizosphere, rhizosphere, and root-surface) community structure to different stumping treatments of sea buckthorn.To address this gap, we hypothesized that: (1) 15 cm stumping would maximize soil nutrient availability and bacterial α-diversity; (2) the abundance of beneficial taxa (*e.g.*, *Oxalobacteraceae*) would peak under 15 cm stumping; (3) shifts in bacterial community composition would be primarily driven by soil nutrient-related parameters rather than physical properties; and (4) stumping treatment effects on overall community structure would be detectable but secondary to spatial heterogeneity. To test these hypotheses, we conducted a field experiment examining soil bacterial communities (non-rhizosphere, rhizosphere, and root-surface) and physicochemical properties under four stumping heights (0, 10, 15, and 20 cm) in a degraded sea buckthorn plantation in the Pisha sandstone region.

## Materials & Methods

### Study area

The study area is located in the Pisha Sandstone Soil and Water Conservation Science and Technology Demonstration Area in Nuanshui Township, Jungar Banner, Ordos City, Inner Mongolia. The geographic coordinates are 39°42′ to 39°50′N latitude and 110°25′ to 110°48′E longitude. This area belongs to a temperate continental monsoon climate, characterized by long, cold winters, short hot summers, and significant temperature fluctuations in spring and autumn. The average annual temperature ranges from 5.3 °C to 7.6 °C. The average frost-free period is 150 days, with an average of 2,981 h of sunshine annually. The average annual precipitation is 400 mm, with the extreme maximum annual rainfall reaching 636.5 mm and the extreme minimum at 143.5 mm. Most of the rainfall occurs in August each year (Huijuan et al., 2024). The primary soil types in the region include loess, chestnut calcareous soil, and wind-sand soil. After native vegetation was severely damaged, the area now largely relies on artificial vegetation cover. The natural vegetation consists mainly of species such as dogwood (*Setaria viridis*), flower stick (*Hedysarum scoparium*), hyacinth (*Achnatherum splendens*), and tamarisk (*Tamarix chinensis*), while artificial vegetation mainly consists of sea buckthorn and mountain almond (*Armeniaca sibirica*). The artificial vegetation is dominated by sea buckthorn, mountain apricot (*Armeniaca sibirica*), and lemon mallow (*Caragana korshinskii*), among others.

### Test methods

In this experiment, a 10-year-old sea buckthorn plantation forest with a plant spacing of 2 m   ×  4 m was selected as the research subject, and stumping treatments were applied. Following the principle of maintaining consistency in standing and growth conditions, treatment plots were set up with stumping heights of 0 cm (A), 10 cm (B), 15 cm (C), and 20 cm (D), along with a control group without stumping (E). Each sample plot covered an area of 50 m × 50 m, with three replicate sample plots for each treatment (Huijuan et al., 2024). In September 2022, soil sampling was conducted. Samples were collected from root surface soil (S), inter-root soil (W), and non-root soil (R). In each sample plot, three healthy sea buckthorn plants with similar plant height and basal diameter were selected for stumping. Surface litter was removed, and soil samples were taken from a depth of 0 to 10 cm, using the ground surface as the reference point. Excess soil around the root system was shaken off manually, leaving about one mm of soil behind. The root system was then cut into small segments of approximately five cm using sterile gloves and sterile scissors, and the samples were packed into 50 mL test tubes and stored in liquid nitrogen. These root samples were later transferred to the laboratory and separated into inter-root and root surface soil, following the procedures described by Gregory Caporaso et al. (2011) and Bolger Anthony, Marc & Bjoern (2014). The collected soil samples were individually placed into sterile soil sample dispensing tubes and stored in a freezer at −70 °C for 16S rDNA sequencing.

### Analysis of soil physical and chemical properties

The methods for analyzing the soil’s physicochemical properties are shown in Table 1 (Huijuan et al., 2024).

### Soil microbial diversity profiling

The structural composition and diversity of the soil bacterial community were determined by Lianchuan Bioinformatics Co (Hangzhou, China).

Soil samples were collected following strict sterile procedures to avoid contamination by exogenous microorganisms. Genomic DNA was extracted using the CTAB method. The purity and concentration of the DNA were assessed by agarose gel electrophoresis (conditions: 1% gel concentration, 100 V, 40 min).

Qualified DNA samples were diluted with sterile water to a concentration of 1 ng/µL to serve as templates. The V4 region of the bacterial 16S rRNA gene was amplified using primers 515F (5′-GTGYCAGCMGCCGCGGTAA-3′) and 806R (5′-GGACTACNVGGGTWTCTAAT-3′) with a high-fidelity DNA polymerase (Gregory Caporaso et al., 2011). The PCR products were checked on a 2% agarose gel, then pooled in equimolar amounts. Target bands were excised and purified using a gel extraction kit. Sequencing libraries were constructed using the TruSeq^®^ DNA PCR-Free Sample Preparation Kit. After quantification with Qubit and validation by Q-PCR, the qualified libraries were sequenced on an Illumina HiSeq 2500 platform (PE250) by LC-Bio Technology Co., Ltd (Hangzhou, China; Gregory Caporaso et al., 2011; Bolger Anthony, Marc & Bjoern, 2014; Reyon et al., 2012).

**Table 1:** Methods for analyzing soil physical and chemical properties.

Norm	Methodologies
Soil moisture content and Soil Bulk Density	Cutting ring method
Soil quick nitrogen	Alkaline diffusion
Soil quick-acting phosphorus	Spectrophotometry
Soil potassium	Flame photometry
Soil organic matter	Potassium dichromate - external heating method

The raw sequencing data were processed using the following bioinformatics pipeline:

Demultiplexing and Initial QC: Samples were demultiplexed based on barcodes. Primer and adapter sequences were trimmed using Cutadapt (v1.9). Paired-end reads were merged using FLASH (v1.2.8) based on their overlap.

Quality Filtering: Sequences were quality-filtered using fqtrim with a sliding window of 100 bp. The 3′ end of a read was truncated when the average quality score within the window fell below 20. Reads shorter than 100 bp after truncation or containing more than 5% ambiguous bases (N) were discarded.

Denoising and ASV Inference: Denoising, chimera removal, and Amplicon Sequence Variant (ASV) calling were performed using the DADA2 plugin within QIIME2. Singleton ASVs (features appearing only once across all samples) were removed by default.

Core ASV Table Construction: To reduce noise from low-abundance sequences, the resulting raw ASV table was filtered. In this study, a core feature table containing 24,501 ASVs was generated and used for all downstream analyses.

Taxonomic Annotation: Representative ASV sequences were taxonomically classified using the feature-classifier classify-sklearn command in QIIME2, against the SILVA 138 reference database, with a confidence threshold set at 0.7.

Data Normalization: To mitigate the effect of uneven sequencing depth on ecological diversity metrics, the ASV abundance table was rarefied (subsampled) to 40,000 sequences per sample using the feature-table rarefy function in QIIME2. This depth was lower than the original sequencing depth of all samples in this dataset (range: 38,573–70,800), ensuring 100% sample retention.

Diversity Analysis: Alpha Diversity: Based on the rarefied data, indices including Shannon, Simpson, Chao1, Good’s coverage, and Pielou’s evenness (Pielou_e) were calculated. Beta Diversity: Beta diversity was assessed based on Bray-Curtis dissimilarity matrices. Principal Coordinate Analysis (PCoA) was performed for visualization, and permutational multivariate analysis of variance (PERMANOVA) was used to test for significant differences between groups.

Differential Species Analysis: Differential species analysis between groups was performed based on normalized relative abundance data. Non-parametric tests were selected according to the experimental design: Fisher’s exact test for comparisons without biological replicates; the Mann–Whitney U test for comparing two groups with replicates; and the Kruskal–Wallis test for multi-group comparisons with replicates. The significance level was set at *p* < 0.05. Prior to downstream analysis, all sequences classified as chloroplast or mitochondrial were removed.

### Data processing

Data were collated using Excel, with one-way analysis of variance (ANOVA) performed using SPSS 26 (IBM Corp., Armonk, NY, USA). Graphing was conducted with Origin 2021, and redundancy analysis (RDA) was performed using Canoco 4.5 software. Data are presented as the mean ± standard deviation. Prior to conducting one-way ANOVA, the underlying assumptions were verified. Specifically, the Shapiro–Wilk test was used to assess normality, and Levene’s test was used to assess the homogeneity of variances. The results confirmed that the data in all groups met the assumptions of normality (all *p* > 0.05) and homogeneity of variances (*p* > 0.05), thus justifying the use of parametric ANOVA. When ANOVA indicated a significant effect, Tukey’s post-hoc test was applied for pairwise comparisons.

To visualize the overall differences in microbial community structure between samples, PCoA was first performed based on the Bray-Curtis distance matrix. Specifically, PCoA was conducted using the ‘cmdscale’ function within the vegan package (version 2.7-2) of R software (version 4.5.2). The coordinates of the first two principal coordinate axes (PCoA1 and PCoA2) were extracted, along with the percentage of total variation explained across samples. Subsequently, scatter plots were generated using the ‘ggplot2’ package (version 4.0.1), with PCoA1 and PCoA2 as axes. Sample points were colored according to different experimental treatment groups to visually demonstrate the clustering of samples within each treatment group and the separation trends between groups. The significance level was set at *p* < 0.05 for all tests.

The comprehensive evaluation was based on the affiliation function method.

The formula is:

If the indicator is positively correlated with plant growth,

(1) \begin{eqnarray*}\mathrm{X} \left( \mathrm{U} \right) = \frac{\mathrm{X}-{\mathrm{X}}_{\mathrm{min}}}{{\mathrm{X}}_{\mathrm{max}}-{\mathrm{X}}_{\mathrm{min}}} .\end{eqnarray*}

If the indicator is negatively correlated with plant growth, the

(2) \begin{eqnarray*}\mathrm{X} \left( \mathrm{U} \right) =1- \frac{\mathrm{X}-{\mathrm{X}}_{\mathrm{min}}}{{\mathrm{X}}_{\mathrm{max}}-{\mathrm{X}}_{\mathrm{min}}} \end{eqnarray*}

where X represents the measured value of the indicator, Xmax is the maximum value of the measurement and Xmin is the minimum value of the measurement.

## Results

### Effects of different stumping treatments on the physico-chemical properties of sea buckthorn soil

The physicochemical properties of sea buckthorn soil following different stumping treatments are summarized in Table 2. Organic matter content was highest with the 15 cm and 10 cm stumping treatments, measuring (21.40 ± 0.95) g/kg and (20.08 ± 0.99) g/kg, respectively. No significant difference was found between these two treatments (*p* > 0.05), though both were significantly higher than the other treatments (*p* < 0.05). This indicates that both 15 cm and 10 cm stumping favor organic matter accumulation.

**Table 2:** Table of physical and chemical properties of *Hippophae rhamnoides* soil after stumping treatment.

The mode of stumping	Organic matter (g/kg)	Quick-acting nitrogen (mg/kg)	Quick-acting phosphorus (mg/kg)	Quick-acting potassium (mg/kg)	Soil moisture content %	Soil capacity (g/cm^3^)
A	18.28 ± 0.16b	18.02 ± 0.24bc	7.043 ± 0.39bc	57.910 ± 0.80c	9.610 ± 0.44bc	1.48 ± 0.01ab
B	20.08 ± 0.99a	18.96 ± 0.60b	7.548 ± 0.49b	62.273 ± 1.39b	10.499 ± 0.27a	1.46 ± 0.02bc
C	21.40 ± 0.95a	20.20 ± 1.09a	8.056 ± 0.82a	64.348 ± 0.72a	9.699 ± 0.41b	1.41 ± 0.02d
D	17.26 ± 0.43b	17.00 ± 0.25c	6.174 ± 0.30c	55.770 ± 0.48c	8.908 ± 0.29bc	1.44 ± 0.01cd
E	14.85 ± 0.10c	13.05 ± 0.69d	4.109 ± 0.44d	49.341 ± 0.36d	8.088 ± 0.45c	1.51 ± 0.01a

**Notes.**

Different lower case letters indicate significant differences between treatments (*P* < 0.05) A indicates full stumping, B indicates 10 cm stumping, C indicates 15 cm stumping, D indicates 20 cm stumping, and E indicates a stumping control group, below.

The maximum concentration of readily available nitrogen (AN) occurred at the 15 cm stumping, at (20.20 ± 1.09) mg/kg, significantly exceeding the other treatments. This demonstrates that the 15 cm stumping markedly enhances the readily AN content. The maximum concentration of readily available phosphorus (AP) was observed at 15 cm (8.056 ± 0.82 mg/kg), significantly exceeding other treatments (*p* < 0.01). This shows that 15 cm stumping most effectively enhances readily available phosphorus. The maximum concentration of readily available potassium (AK) was also observed at 15 cm stumping, at (64.348 ± 0.72) mg/kg, significantly higher than in other treatments (*p* < 0.05).

The highest soil moisture content (SMC), was recorded at the 10 cm stumping height, at (10.499 ± 0.27%), significantly higher than in all other treatments (*p* < 0.05). This indicates that a 10 cm stumping significantly increased SMC, with the 15 cm stumping also showing some improvement. The highest soil bulk density (BD) occurred in the control group (1.41 g/cm^3^), significantly higher than in the 15 cm and 20 cm stumping treatments (*p* < 0.05), but not significantly different from full stumping removal or the 10 cm stumping treatment (*p* > 0.05). This indicates that the control group exhibited the highest BD, while the 15 cm stumping group showed the lowest, suggesting a looser soil structure under the 15 cm stumping treatment.

In summary, the 15 cm stumping produced the most pronounced improvements in soil properties.

### Analysis of the number of ASVs in samples under different stumping treatments

**Figure 1:**
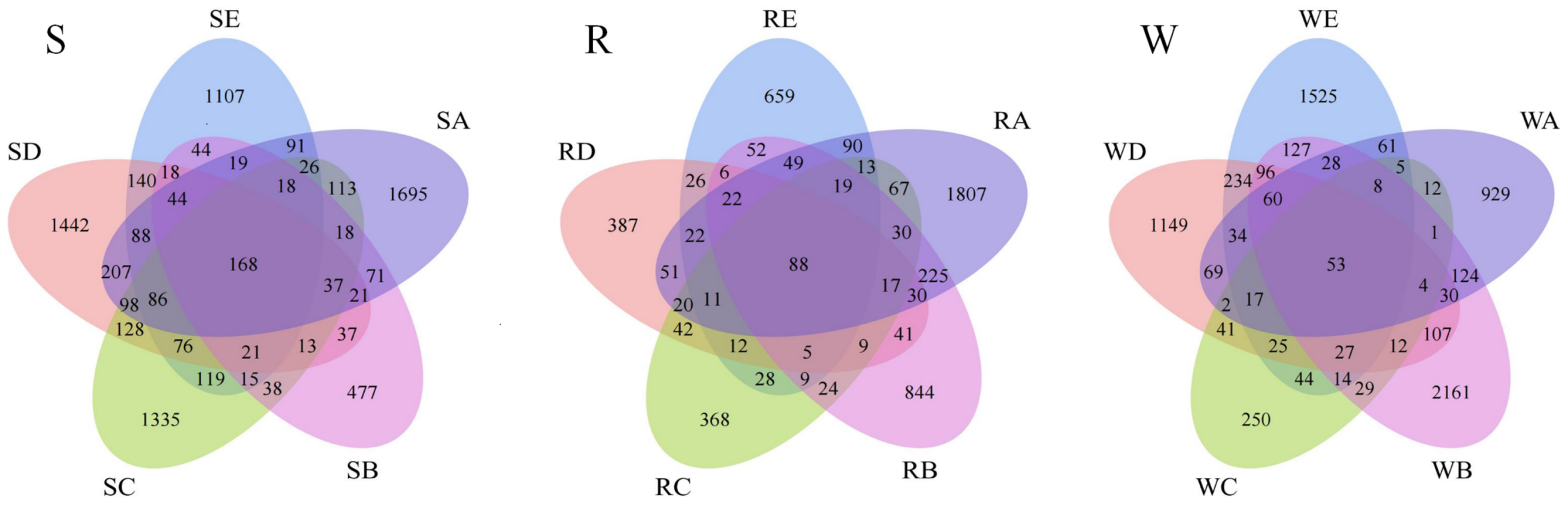
Venn diagram of ASV distribution across surface soil (S) non-rhizosphere soil (R), and rhizosphere soil (W).

To demonstrate the number of shared *versus* unique Amplicon Sequence Variants (ASVs) between individual samples or subgroups, we visualized this using a Venn diagram. In the Venn diagram shown in Fig. 1, each circle represents a stumping treatment, and the overlap between the circles indicates the number of ASVs shared between different treatments, while the non-overlapping part shows the number of ASVs unique to each treatment.

 For the R (root surface soil), a total of 6,693 ASVs were identified across the five stumping treatments, with 88 ASVs shared between treatments. The number of unique ASVs was 639, 387, 368, 844, and 1,807 for each treatment, respectively. For the W (rhizosphere soil), a total of 9,180 ASVs were identified, with 53 co-owned ASVs, and unique ASVs numbered 1,525, 1,149, 250, 2,161, and 929, respectively. For the S (non-rhizosphere soil), 10,872 ASVs were identified, with 168 co-owned ASVs and 1,107, 1,442, 1,335, 477, and 1,695 unique ASVs, respectively.

The percentage of shared ASVs was as follows: R: 1.31%, W: 0.58%, S: 1.55%.

The percentage of unique ASVs was as follows: R-specific ASVs: 9.55%, 5.77%, 5.50%, 12.61%, and 27.00%, W-specific ASVs: 16.61%, 16.61%, 16.52%, 5.10%, 12.52%, 5.10%, 23.54%, and 10.12%. S-specific ASVs: 10.18%, 13.26%, 12.28%, 4.39%, and 15.59%

The bacterial ASV ratios for the unlevelled and fully levelled stumping soils were approximately: R: 0.43, W: 1.64, S: 0.74. 10 cm stumping: R: 0.75, W: 0.82, S: 1.96. 15 cm stumping: R: 1.45, W: 5.33, S: 0.90. 20 cm stumping: R: 1.40, W: 1.20, S: 0.79.

The results indicate that 10 cm stumping primarily affected bacterial ASVs in the root surface soil, while 15 cm stumping had a more significant effect on species composition at the ASV level in both the non-root surface soil and root surface soil.

### Analysis of soil bacterial community structure α-diversity under different stumping treatments

According to the results of the α-diversity index, as shown in Table 3, Good’s coverage values were 1.00 for all samples, indicating that the sequencing depth captured the full bacterial diversity. The Chao1 index, which estimates the total number of species in a community, including those potentially not observed, was also used to assess species richness. In the case of R, the highest number of species was observed in the 15 cm stumping treatment, followed by the 10 cm stumping treatment; for W, the highest species richness was found in the 15 cm stumping treatment, followed by the 0 cm stumping treatment; and for S, the highest species richness occurred in the 15 cm stumping treatment, followed by the 10 cm stumping treatment.

**Table 3:** Microbial diversity index analysis of soil-root continuum samples.

	Shannon	Simpson	Chao1	Goods_coverage	Pielou_e
RA	6.25 ± 0.27b	0.93 ± 0.03a	902.67 ± 68.66d	1 ± 0.00a	0.67 ± 0.07a
RB	6.60 ± 0.18b	0.90 ± 0.06a	1,439.33 ± 25.52b	1 ± 0.00a	0.69 ± 0.14a
RC	7.69 ± 0.10a	0.93 ± 0.05a	1,643.11 ± 69.43a	1 ± 0.00a	0.69 ± 0.15a
RD	6.31 ± 0.16b	0.92 ± 0.04a	1,084.61 ± 21.98c	1 ± 0.00a	0.66 ± 0.11a
RE	5.52 ± 0.40c	0.92 ± 0.03a	814.66 ± 64.22d	1 ± 0.00a	0.65 ± 0.07a
WA	8.37 ± 0.25a	0.91 ± 0.08a	1,516.41 ± 221.80b	1 ± 0.00a	0.66 ± 0.21a
WB	8.50 ± 0.21a	0.92 ± 0.07a	1,465.03 ± 99.72b	1 ± 0.00a	0.65 ± 0.17a
WC	8.76 ± 0.20a	0.98 ± 0.01a	1,853.10 ± 93.56a	1 ± 0.00a	0.82 ± 0.05a
WD	7.52 ± 0.21b	0.92 ± 0.07a	1,004.45 ± 78.51c	1 ± 0.00a	0.66 ± 0.16a
WE	5.69 ± 0.38c	0.90 ± 0.03a	791.77 ± 57.94d	1 ± 0.00a	0.58 ± 0.06a
SA	8.26 ± 0.25b	0.97 ± 0.04a	1,681.51 ± 139.49c	1 ± 0.00a	0.78 ± 0.11a
SB	8.52 ± 0.22b	0.94 ± 0.07a	2,114.20 ± 29.55b	1 ± 0.00a	0.72 ± 0.17a
SC	9.80 ± 0.13a	0.99 ± 0.02a	2,432.13 ± 156.57a	1 ± 0.00a	0.84 ± 0.06a
SD	8.28 ± 0.27b	0.97 ± 0.04a	1,734.25 ± 85.79c	1 ± 0.00a	0.78 ± 0.12a
SE	7.34 ± 0.33c	0.93 ± 0.06a	1,008.92 ± 44.12d	1 ± 0.00a	0.70 ± 0.17a

The Shannon index measures diversity, with higher values indicating higher diversity. The Shannon index was highest for R, W and S in the 15 cm stumping treatment, suggesting that this treatment enhances microbial diversity.

The Simpson index ranges from 0 to 1, with a value of 0 indicating the lowest diversity (only one species in the community) and a value of one indicating the highest diversity (extremely rich species with an even distribution). In this study, the Simpson index for most of the samples ranged between 0.8 and 1.0, indicating a high degree of evenness in the distribution of species.

Pielou’s index, which measures the evenness of species distribution (the higher the value, the more uniform the distribution), showed that S had the highest evenness compared to R and W, indicating that S had the highest Pielou index in the 15 cm stumping treatment.

### Analysis of soil bacterial community structure β-Diversity under different stumping treatments

To analyze the effects of different treatments and spatial location (the three plots R, S, and W) on microbial community structure, we conducted a multivariate PERMANOVA analysis based on the Bray–Curtis distance matrix (permutations = 999). The results indicated that the overall model incorporating both block and treatment factors was significant (Pseudo-F = 1.76, *P* = 0.014). Further decomposition of factor effects revealed that differences among the three plots (R, S, and W) had a highly significant influence on community variation (*R*^2^ = 11.89%, *P* = 0.008), indicating substantial differences in community composition between the R, S, and W blocks. In contrast, after controlling for these plot-to-plot differences, differences between treatments did not reach statistical significance (*R*^2^ = 9.89%, *P* = 0.226). Additionally, the PERMDISP test for homogeneity of dispersion between groups was non-significant (*P* = 0.833), confirming the reliability of the PERMANOVA results and ruling out variation within groups as an explanatory factor.

**Figure 2:**
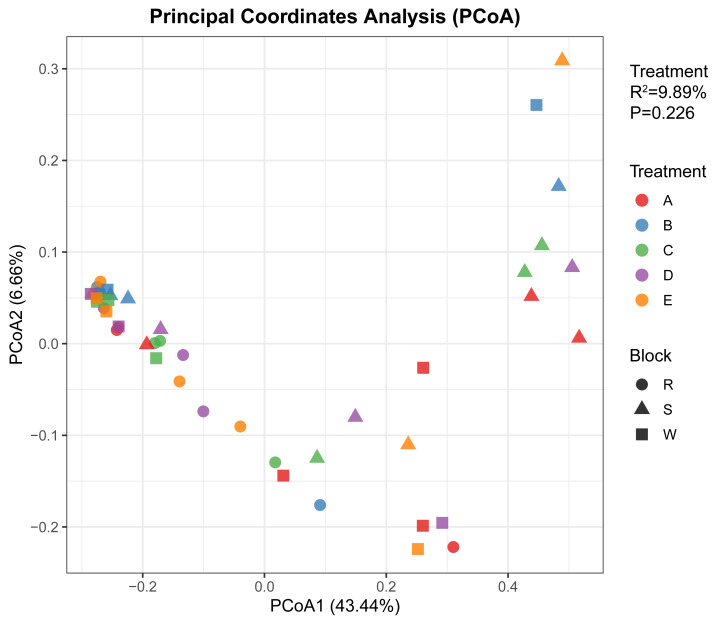
PCoA analysis of microbial diversity in soil bacterial samples.

Visualization based on Principal Coordinate Analysis (PCoA) (Fig. 2) corroborated these statistical findings. The PCoA1 and PCoA2 axes explained 43.44% and 6.66% of the community variation, respectively. The plot reveals that sample points first form distinct clusters based on plot block (represented by different shapes). For instance, all samples from Plot Block R tend to cluster in a specific region, visually demonstrating that the plot block effect is the primary driver of community variation. Simultaneously, sample points from different treatments (denoted by distinct colors) exhibit spatially intermingled distributions without forming independent clusters. This aligns with the statistically insignificant treatment effects. *Post-hoc* pairwise comparisons also failed to identify any significant differences between treatment groups (all corrected *P* > 0.05).

### Analysis of the bacterial community structure of sea buckthorn soil under different stumping treatments

**Figure 3:**
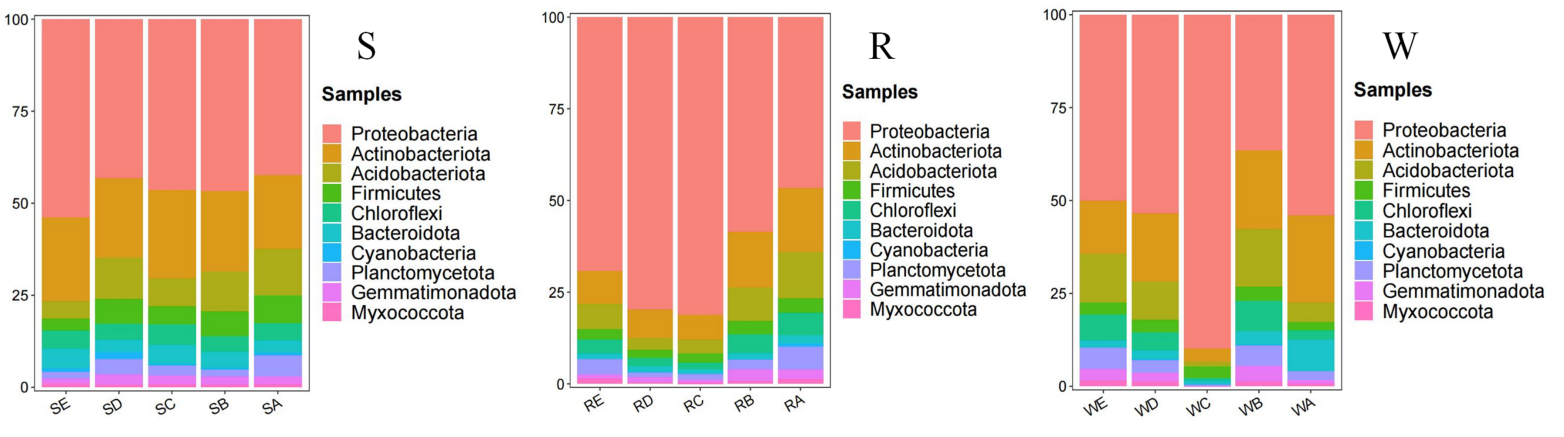
SRW relative abundance at the bacterial phylum level.

Soil bacterial communities exhibited high diversity across all stumping treatments, as indicated by the Shannon diversity index (ranging from 5.69 to 9.80) and Chao1 richness index (ranging from 791.77 to 2,432.13) (Table 3). The 15 cm stumping treatment consistently showed the highest values for both indices across all soil compartments. At the phylum level (Fig. 3), the top ten dominant bacterial phyla in terms of relative abundance were *Proteobacteria*, *Actinobacteriota*, *Acidobacteriota*, *Firmicutes*, *Chloroflexi*, *Bacteroidota*, *Cyanobacteria*, *Globacteria*, and *Bacteriophage*, including *Bacteroidota*, *Cyanobacteria*, *Planctomycetota*, *Gemmatimonadota*, and *Myxococcota*. The relative abundance of *Proteobacteria* was the highest across all treatments and soils, with the highest relative abundance observed in R and W in the 15 cm stumping treatment. The proportions of *Proteobacteria* and *Actinomycetes* were higher in S, showing minimal variation across different stumping treatments.

**Figure 4:**
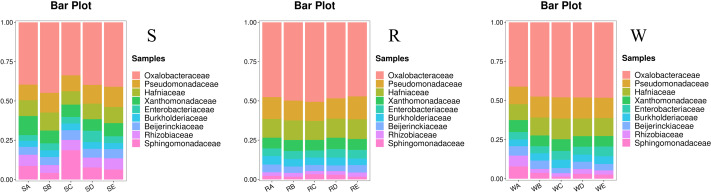
SRW relative abundance at the level of the family.

At the family level, all treatments exhibited highly consistent community structure characteristics (Fig. 4). *Oxalobacteraceae*, *Pseudomonadaceae*, and *Hafniaceae* stably dominated across the three regions, forming the core microbiota. Among these, *Oxalobacteraceae* had the highest relative abundance, generally ranging between 20% and 40%. Further comparisons revealed reproducible microbial enrichment patterns for the same treatment across regions. For example, the 15 cm stumping treatment (including RC, SC, and WC) most significantly enriched *Oxalobacteraceae*, with its abundance peaking at 38.9% in the WC treatment. In contrast, complete stumping (including RA, SA, and WA) generally resulted in lower abundances of dominant families, such as *Oxalobacteraceae*, which only reached 15.2% in the WA treatment.

Notably, data from the S region exhibited relatively high within-group variation. For example, the abundance of *Oxalobacteraceae* in the SB treatment ranged from 4.1% to 40.7%, reflecting the inherent spatial heterogeneity of soil microorganisms in the field. Nevertheless, the consistent treatment effects and stable core microbiota composition indicate that stumping is a key driver shaping soil bacterial community structure.

### Relationship between bacterial dominant phylum and soil physico-chemical properties of sea buckthorn soil under different stumping treatments

**Figure 5:**
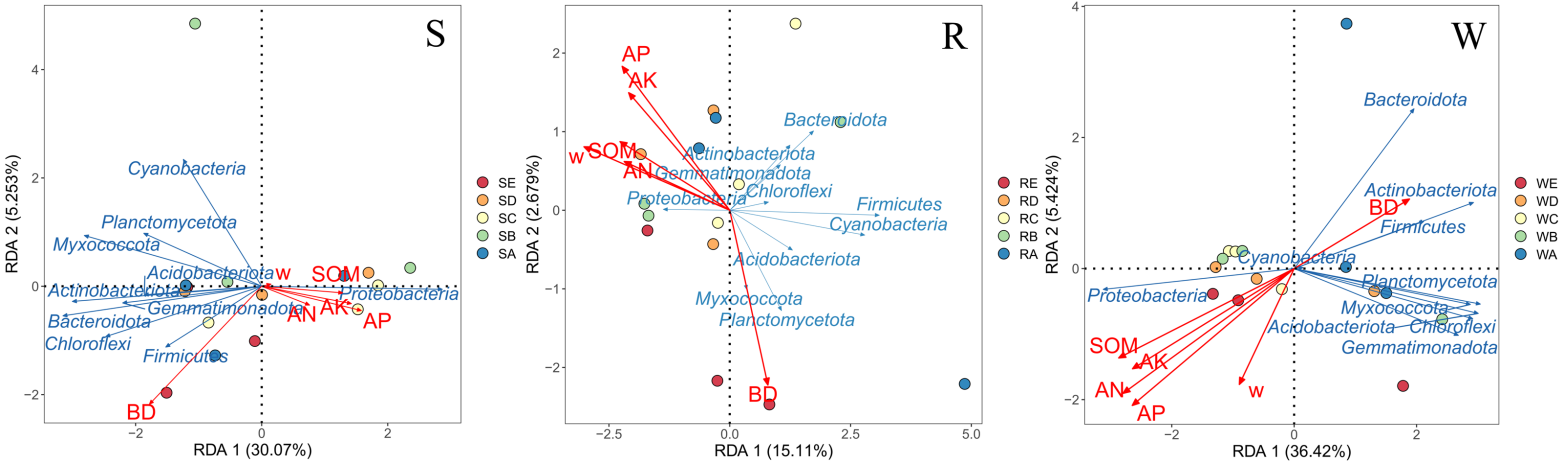
RDA analysis of soil root continuum and soil physicochemical properties.

To investigate the influence of environmental factors and soil bacterial communities, we conducted an Redundancy Analysis (RDA) of soil physicochemical properties and bacterial community composition at the phylum level. The results of this analysis are shown in Fig. 5. The AP, AK, and BD rays were longer at the phylum level in S, indicating that these factors had a greater influence on the structure of the bacterial community at the phylum level. Similarly, AP and AN showed longer vectors in the W ordination, suggesting that they explained more variation in bacterial community composition compared to other factors.

In S, W, and R, AN, soil organic matter (SOM), AP, AK, and w were positively correlated with the *Actinobacteriota* phylum, while BD was negatively correlated with it. These results suggest that soil microorganisms and soil physicochemical properties under different stumping treatments have a mutual influence on each other.

### Comprehensive analysis of sea buckthorn characteristics under different stumping treatments

In this study, we comprehensively analyzed and evaluated the indices of sea buckthorn soil and microorganisms under different stumping treatments using the affiliation function method. The indicators of sea buckthorn, namely: soil organic matter (X1), quick-acting phosphorus (X2), quick-acting nitrogen (X3), quick-acting potassium (X4), soil water content (X5), soil bulk density (X6), number of ASVs (X7), Shannon index (X8), Simpson index (X9), Chao1 index (X10), and Pielou’s evenness (X11), were used for comprehensive evaluation.

As shown in Tables 4–6, the affiliation function values for the indicators of soil microorganisms in sea buckthorn under different stumping leveling modes were highest in the 15 cm stumping leveling for root surface soil, inter-root soil, and non-inter-root soil.

## Discussion

Soil physicochemical properties are fundamental drivers of bacterial community structure (Jing et al., 2016; Yanfa et al., 2016; Nan et al., 2020). Our RDA revealed that AN, SOM, AP, and AK were the primary edaphic factors shaping bacterial communities under different stumping treatments. These nutrient-related parameters showed consistent positive correlations with most dominant phyla, particularly with *Actinobacteriota*, which exhibited strong positive associations with SOM, AN, AP, AK, and SWC, and a negative correlation with BD. This aligns with Chunying et al. (2021) and Guohui et al. (2020), who reported similar linkages between microbial composition and soil nutrient status. The marked influence of nutrient availability suggests that stumping primarily affects microbial communities through resource-mediated pathways, rather than *via* direct physical disturbance.

Among all treatments, 15 cm stumping consistently produced the strongest improvements in soil organic carbon, available nutrients, and bacterial α-diversity (Chao1, Shannon). This pattern likely reflects an optimal resource balance: 15 cm stumping stimulates root exudation and fine root turnover without causing excessive carbon limitation or water stress, thereby enhancing both nutrient pools and microbial richness. In contrast, 20 cm stumping or complete stumping removal may reduce plant carbon input, while insufficient stumping (0 or 10 cm) fails to sufficiently rejuvenate root activity (Haoxia, Xuefei & Ying, 2022; Xiyang et al., 2020; Xia et al., 2021). These findings support our hypothesis that moderate disturbance optimizes soil–microbe feedbacks in degraded plantations.

**Table 4:** Comprehensive evaluation of root topsoil membership function method under different stumping mode.

Mode	X1	X2	X3	X4	X5	X6	X7	X8	X9	X10	X11	Average value
SA	0.47	0.47	0.43	0.63	0.55	0.50	0.47	0.43	0.62	0.48	0.55	0.51
SB	0.48	0.47	0.42	0.61	0.53	0.80	0.45	0.58	0.36	0.44	0.35	0.50
SC	0.48	0.47	0.43	0.61	0.58	0.75	0.59	0.36	0.67	0.38	0.64	0.54
SD	0.43	0.43	0.41	0.55	0.61	0.67	0.51	0.46	0.57	0.55	0.53	0.52
SE	0.42	0.46	0.54	0.46	0.57	0.50	0.66	0.42	0.62	0.40	0.35	0.49

**Table 5:** Comprehensive evaluation of rhizosphere soil membership function method under different stumping modes.

Mode	X1	X2	X3	X4	X5	X6	X7	X8	X9	X10	X11	Average value
SA	0.47	0.47	0.43	0.63	0.55	0.50	0.42	0.61	0.50	0.42	0.63	0.51
SB	0.48	0.47	0.42	0.61	0.53	0.67	0.35	0.58	0.40	0.54	0.61	0.51
SC	0.48	0.47	0.43	0.61	0.58	0.75	0.36	0.58	0.40	0.45	0.72	0.53
SD	0.43	0.43	0.41	0.55	0.61	0.67	0.41	0.61	0.46	0.36	0.60	0.50
SE	0.42	0.46	0.54	0.46	0.57	0.50	0.35	0.46	0.46	0.58	0.64	0.49

**Table 6:** Comprehensive evaluation of non-rhizosphere soil membership function method under different stumping modes.

Mode	X1	X2	X3	X4	X5	X6	X7	X8	X9	X10	X11	Average value
RA	0.47	0.47	0.43	0.63	0.55	0.50	0.34	0.44	0.33	0.54	0.64	0.48
RB	0.48	0.47	0.42	0.61	0.53	0.67	0.34	0.63	0.33	0.46	0.41	0.49
RC	0.48	0.47	0.43	0.61	0.58	0.75	0.29	0.49	0.37	0.57	0.65	0.52
RD	0.43	0.43	0.41	0.55	0.61	0.67	0.59	0.44	0.62	0.44	0.25	0.49
RE	0.42	0.46	0.54	0.46	0.57	0.50	0.59	0.44	0.60	0.41	0.33	0.48

At the family level, *Oxalobacteraceae*, *Pseudomonadaceae*, and *Hafniaceae* formed a stable core microbiota across all soil compartments. Notably, 15 cm stumping most strongly enriched *Oxalobacteraceae*, with relative abundance peaking at 38.9% in rhizosphere soil. Members of *Oxalobacteraceae* are known copiotrophs that respond rapidly to increased nutrient availability and are often associated with plant growth promotion and suppression of soil-borne pathogens (Spain, Krumhol & Elshahed, 2009; Lan et al., 2019). Their consistent enrichment under 15 cm stumping suggests that this practice not only improves soil fertility but also enhances the functional potential of the rhizosphere microbiome. The high within-group variation observed in non-rhizosphere soil (*e.g.*, *Oxalobacteraceae* abundance ranging from 4.1% to 40.7% in the SB treatment) reflects the inherent spatial heterogeneity of field soils, yet the reproducible treatment effects underscore the ecological relevance of stumping as a management tool.

Although stumping significantly altered individual taxa and α-diversity, β-diversity was primarily governed by spatial heterogeneity among plots rather than treatment itself. PERMANOVA confirmed that differences among plots explained a significant portion of community variation (*P* = 0.008), whereas treatment effects were non-significant after accounting for spatial structure (*P* = 0.226). This result is consistent with studies showing that pre-existing soil gradients often override the effects of short-term management interventions (Hui et al., 2023). The non-significant PERMDISP test (*P* = 0.833) further confirms that the lack of a treatment effect on community composition is genuine and not an artifact of dispersion heterogeneity. Thus, stumping effects on overall community structure are superimposed on, rather than replacing, the strong natural heterogeneity of the Pisha sandstone soils.

At the phylum level, *Proteobacteria*, *Actinobacteriota*, and *Acidobacteriota* dominated across all treatments, consistent with their cosmopolitan distribution in terrestrial ecosystems (Zhen & Chendi, 2021; Chaudhry et al., 2012). The predominance of *Proteobacteria*—a phylum often linked to carbon mineralization and plant growth promotion—supports the notion that stumping maintains or enhances soil functional capacity (Chaudhry et al., 2012; Fazi et al., 2005). *Actinobacteriota* are known to participate in organic matter decomposition (Fazi et al., 2005), and their positive correlation with multiple nutrient indicators further implies that this phylum plays a key role in nutrient cycling under improved soil conditions. Negassa et al. found that *Actinobacteriota* and *Proteobacteria* primarily inhabit macroporous spaces, while *Acidobacteriota* are more prevalent in small pore spaces; conversely, microbial metabolic activity can also modify soil pore structure (Zhiyong, 2022). This bidirectional interaction has positive implications for improving soil structure in the Pisha sandstone region. Thus, the observed shifts in *Actinobacteriota* and *Proteobacteria* under 15 cm stumping may not only reflect improved nutrient availability but could also contribute to long-term improvements in soil physical structure, creating a positive feedback loop between soil properties and microbial communities.

Taken together, our results demonstrate that 15 cm stumping effectively enhances soil nutrient status, bacterial diversity, and the abundance of beneficial taxa, with the most pronounced effects occurring in root-associated soils. However, these effects are context-dependent and modulated by inherent spatial variability. Future studies should integrate metagenomic or metatranscriptomic approaches to resolve the functional gene responses underpinning the observed taxonomic shifts (Lan et al., 2019), and long-term field trials are needed to assess the sustainability of stumping-mediated soil improvements. Such knowledge will aid in refining stumping regimes for soil conservation and plantation restoration in the Pisha sandstone region and analogous eroded landscapes.

## Conclusions

This study investigated the effects of different stumping heights on soil physicochemical properties and bacterial communities in a degraded sea buckthorn plantation in the Pisha sandstone region. Our results demonstrate that 15 cm stumping consistently outperformed other treatments, significantly improving soil organic carbon, available nutrients, and bacterial α-diversity, while also enriching beneficial taxa such as Oxalobacteraceae (peaking at 38.9% in rhizosphere soil).

At the community level, β-diversity variation was primarily driven by spatial heterogeneity among experimental plots (*R*^2^ = 11.89%, *P* = 0.008), with stumping treatment effects being detectable but secondary (*R*^2^ = 9.89%, *P* = 0.226). Redundancy analysis further revealed that *Actinobacteriota* abundance was positively correlated with nutrient-related soil parameters (organic matter, available N, P, K, and moisture) and negatively correlated with BD, suggesting that stumping influences microbial communities mainly through resource-mediated pathways.

Membership function analysis confirmed that 15 cm stumping yielded the highest comprehensive scores across all soil compartments, particularly in root-associated soils. Collectively, these findings provide a microbial perspective for optimizing stumping regimes in soil erosion control and plantation restoration. Future studies should investigate the functional gene responses to stumping and explore the long-term dynamics of key microbial taxa under different management practices.

##  Supplemental Information

10.7717/peerj.21234/supp-1 Supplemental Information 1 Data Volume 1

10.7717/peerj.21234/supp-2 Supplemental Information 2 Data Volume 2

## Additional Information and Declarations

### Data Availability

The following information was supplied regarding data availability: The raw data is available in the Supplemental Files. The high-throughput data is available at NCBI: PRJNA1302650.
