# Supplementary material for: Response of soil bacterial community structure in Pisha sandstone areas to different stumping
Source: PeerJ. 2026 May 4;14:e21234. doi: 10.7717/peerj.21234 (PMC13151931; doi:10.7717/peerj.21234)
Supplement: Supplemental Information 2 [file peerj-14-21234-s002.z01 › Data/DATA (2)/Response letter.docx]

Dear Respected Editor,

I hope this message finds you well.

Thank you very much for your kind guidance and for granting us 14 days to revise our manuscript (ID: [122571], entitled 《Response of soil bacterial community structure in Pisha sandstone areas to different stumping》). We sincerely appreciate the time and effort you and the reviewers have dedicated to improving our work.

We are pleased to upload our revised manuscript through the online submission system. Out of the utmost respect for your time and to avoid any delay in the editorial process, we have worked diligently to complete the revisions as quickly as possible. All changes have been clearly marked in the "tracked changes" version for your convenient review.

Below is our point-by-point response to the reviewers' comments:

**Reviewer 1:**

**Comment:** The revision is greatly improved. There are only minor instances of missing capitalized letters, etc. These can be fixed during copy editing.

**Response:** We sincerely thank Reviewer 1 for the positive feedback and are glad that the revisions meet with approval. We will ensure that any remaining minor formatting issues are addressed during the proofreading stage.

**Reviewer 3:**

**Comment 1:** The language of the manuscript should be corrected and streamlined by removing vague or unclear terms. For example, in line 404 the phrase "severe stumping" is not clearly defined. Authors should provide clear and reasonable names for the stumping treatments and then use these terms consistently throughout the manuscript to avoid confusion.

**Response:** We thank Reviewer 3 for this valuable suggestion. To eliminate ambiguity, we have now replaced all subjective terms (such as "moderate," "severe," stumping) with specific stumping heights throughout the manuscript.

**For example:**

"moderate stumping" → "15 cm stumping"

"severe stumping" → "20 cm stumping"

**Comment 2:** Line 322 states: "Soil microbial diversity was highly rich under different stumping treatments." The term "highly rich" is vague. Authors should describe the results with clearer and more precise wording.

**Response:** We agree with the reviewer that "highly rich" was imprecise. We have now replaced this phrase with a more specific description based on our diversity indices (Lines 324-327):

"Soil bacterial communities exhibited high diversity across all stumping treatments, as indicated by the Shannon diversity index (ranging from 5.69 to 9.80) and Chao1 richness index (ranging from 791.77 to 2432.13) (Table 3). The 15 cm stumping treatment consistently showed the highest values for both indices across all soil compartments."

**Comment 3:** Lines 355/321 versus lines 295/246: Some section headings begin with capital letters while others do not. Please make the formatting of headings consistent throughout the manuscript.

**Response:** We have carefully reviewed all section headings throughout the manuscript and ensured that they now follow a consistent Title Case format (i.e., each major word capitalized). The specific headings mentioned have been corrected accordingly.

**Comment 4:** Line 420: Authors should use clear and appropriate terms and avoid statements that are not explained. For example, the term "block effects" is mentioned but not clearly defined. Authors should clarify what is meant by this term.

**Response:** We thank the reviewer for this important clarification. To avoid any ambiguity, we have removed the term "block effects" entirely and replaced it with clear, self-explanatory descriptions throughout the manuscript.

**For example:**

In the Results section: "differences among the three plots (R, S, and W)"

In the Discussion section: "spatial heterogeneity among plots"

In the Conclusions section: "spatial heterogeneity among experimental plots"

All statistical results (R²and P values) remain unchanged and are now presented alongside these clarified descriptions.

In addition, following your recent email instructions, we have also addressed the technical points below:

| **Request** | **Action Taken** |
| --- | --- |
| 1. Language in Data File(s) | We have translated all Chinese terms (e.g., "根内") in the Data 148.xlx file into English. An English-language codebook has also been provided for clarity. |
| 2. Clean Manuscript Source File | We have accepted all tracked changes and turned off revision mode. The clean, unmarked source file (Manuscript_Clean.docx) is now uploaded. |
| 3. Broken Reference (Line 178) | We have thoroughly checked the entire manuscript by displaying all field codes (Alt+F9) and searching for keywords such as "Error" and "Reference source not found". No such error was found in the current version. We suspect that the issue may have been from an earlier draft and has been resolved during the revision process. All cross-references have been updated and are functioning correctly. |

Could I be so bold as to ask for your kind understanding regarding a personal matter? The first author, Lu Cheng, is facing a critical deadline for graduation and is required to obtain the official publication "检索证明" (i.e., a certificate from the library confirming that the article has been indexed in Web of Science, Scopus, or similar databases) by April 16, 2026. If the revised manuscript is found to be acceptable, we would be immeasurably grateful if the editorial office could process it at your earliest convenience. We fully understand that you have a very busy schedule with many manuscripts to handle, and we sincerely apologize for any trouble this request may cause. Please accept this not as a demand, but as a humble plea from a student whose entire graduation depends on this outcome.

Thank you once again from the bottom of our hearts for your time, your understanding, and the tremendous effort you and the reviewers have invested in our manuscript. We are truly grateful for your consideration.

With warm regards and deepest respect,

Sincerely,

Lu Cheng

On behalf of all authors
