# Supplementary material for: Response of soil bacterial community structure in Pisha sandstone areas to different stumping
Source: PeerJ. 2026 May 4;14:e21234. doi: 10.7717/peerj.21234 (PMC13151931; doi:10.7717/peerj.21234)
Supplement: Supplemental Information 2 [file peerj-14-21234-s002.z01 › Data/DATA (2)/Table/Data136.docx]

Table 1 Methods for Analyzing Soil Physical and Chemical Properties

| norm | methodologies |
| --- | --- |
| Soil moisture content and Soil Bulk Density | cutting ring method |
| Soil quick nitrogen | alkaline diffusion |
| Soil quick-acting phosphorus | spectrophotometry |
| Soil potassium | flame photometry |
| Soil organic matter | Potassium dichromate - external heating method |
