# Supplementary material for: Response of soil bacterial community structure in Pisha sandstone areas to different stumping
Source: PeerJ. 2026 May 4;14:e21234. doi: 10.7717/peerj.21234 (PMC13151931; doi:10.7717/peerj.21234)
Supplement: Supplemental Information 2 [file peerj-14-21234-s002.z01 › Data/DATA (2)/Table/Data137.docx]

Table 2 Table of physical and chemical properties of *Hippophae rhamnoides* soil after stumping treatment

| The mode of stumping | organic matter(g/kg) | quick-acting nitrogen(mg/kg) | quick-acting phosphorus (mg/kg) | quick-acting potassium(mg/kg) | soil moisture content% | soil capacity  (g/cm³) |
| --- | --- | --- | --- | --- | --- | --- |
| A | 18.28±0.16b | 18.02±0.24bc | 7.043±0.39bc | 57.910±0.80c | 9.610±0.44bc | 1.48±0.01ab |
| B | 20.08±0.99a | 18.96±0.60b | 7.548±0.49b | 62.273±1.39b | 10.499±0.27a | 1.46±0.02bc |
| C | 21.40±0.95a | 20.20±1.09a | 8.056±0.82a | 64.348±0.72a | 9.699±0.41b | 1.41±0.02d |
| D | 17.26±0.43b | 17.00±0.25c | 6.174±0.30c | 55.770±0.48c | 8.908±0.29bc | 1.44±0.01cd |
| E | 14.85±0.10c | 13.05±0.69d | 4.109±0.44d | 49.341±0.36d | 8.088±0.45c | 1.51±0.01a |

Note: Different lower case letters indicate significant differences between treatments (P<0.05) A indicates full stumping, B indicates 10cm stumping, C indicates 15cm stumping, D indicates 20cm stumping, and E indicates a stumping control group, below.
