# Supplementary material for: Response of soil bacterial community structure in Pisha sandstone areas to different stumping
Source: PeerJ. 2026 May 4;14:e21234. doi: 10.7717/peerj.21234 (PMC13151931; doi:10.7717/peerj.21234)
Supplement: Supplemental Information 2 [file peerj-14-21234-s002.z01 › Data/DATA (2)/Table/Data138.docx]

Table 3 Microbial diversity index analysis of soil-root continuum samples

|  | shannon | simpson | chao1 | goods_coverage | pielou_e |
| --- | --- | --- | --- | --- | --- |
| RA | 6.25±0.27b | 0.93±0.03a | 902.67±68.66d | 1±0.00a | 0.67±0.07a |
| RB | 6.60±0.18b | 0.90±0.06a | 1439.33±25.52b | 1±0.00a | 0.69±0.14a |
| RC | 7.69±0.10a | 0.93±0.05a | 1643.11±69.43a | 1±0.00a | 0.69±0.15a |
| RD | 6.31±0.16b | 0.92±0.04a | 1084.61±21.98c | 1±0.00a | 0.66±0.11a |
| RE | 5.52±0.40c | 0.92±0.03a | 814.66±64.22d | 1±0.00a | 0.65±0.07a |
| WA | 8.37±0.25a | 0.91±0.08a | 1516.41±221.80b | 1±0.00a | 0.66±0.21a |
| WB | 8.50±0.21a | 0.92±0.07a | 1465.03±99.72b | 1±0.00a | 0.65±0.17a |
| WC | 8.76±0.20a | 0.98±0.01a | 1853.10±93.56a | 1±0.00a | 0.82±0.05a |
| WD | 7.52±0.21b | 0.92±0.07a | 1004.45±78.51c | 1±0.00a | 0.66±0.16a |
| WE | 5.69±0.38c | 0.90±0.03a | 791.77±57.94d | 1±0.00a | 0.58±0.06a |
| SA | 8.26±0.25b | 0.97±0.04a | 1681.51±139.49c | 1±0.00a | 0.78±0.11a |
| SB | 8.52±0.22b | 0.94±0.07a | 2114.20±29.55b | 1±0.00a | 0.72±0.17a |
| SC | 9.80±0.13a | 0.99±0.02a | 2432.13±156.57a | 1±0.00a | 0.84±0.06a |
| SD | 8.28±0.27b | 0.97±0.04a | 1734.25±85.79c | 1±0.00a | 0.78±0.12a |
| SE | 7.34±0.33c | 0.93±0.06a | 1008.92±44.12d | 1±0.00a | 0.70±0.17a |
