# Supplementary material for: Response of soil bacterial community structure in Pisha sandstone areas to different stumping
Source: PeerJ. 2026 May 4;14:e21234. doi: 10.7717/peerj.21234 (PMC13151931; doi:10.7717/peerj.21234)
Supplement: Supplemental Information 2 [file peerj-14-21234-s002.z01 › Data/DATA (2)/Table/Data139.docx]

Table 4 Comprehensive evaluation of root topsoil membership function method under different flat mode

| Mode | X1 | X2 | X3 | X4 | X5 | X6 | X7 | X8 | X9 | X10 | X11 | average value |
| --- | --- | --- | --- | --- | --- | --- | --- | --- | --- | --- | --- | --- |
| SA | 0.47 | 0.47 | 0.43 | 0.63 | 0.55 | 0.50 | 0.47 | 0.43 | 0.62 | 0.48 | 0.55 | 0.51 |
| SB | 0.48 | 0.47 | 0.42 | 0.61 | 0.53 | 0.80 | 0.45 | 0.58 | 0.36 | 0.44 | 0.35 | 0.50 |
| SC | 0.48 | 0.47 | 0.43 | 0.61 | 0.58 | 0.75 | 0.59 | 0.36 | 0.67 | 0.38 | 0.64 | 0.54 |
| SD | 0.43 | 0.43 | 0.41 | 0.55 | 0.61 | 0.67 | 0.51 | 0.46 | 0.57 | 0.55 | 0.53 | 0.52 |
| SE | 0.42 | 0.46 | 0.54 | 0.46 | 0.57 | 0.50 | 0.66 | 0.42 | 0.62 | 0.40 | 0.35 | 0.49 |
