# Supplementary material for: Response of soil bacterial community structure in Pisha sandstone areas to different stumping
Source: PeerJ. 2026 May 4;14:e21234. doi: 10.7717/peerj.21234 (PMC13151931; doi:10.7717/peerj.21234)
Supplement: Supplemental Information 2 [file peerj-14-21234-s002.z01 › Data/DATA (2)/Text-only manuscript-Response of soil bacterial community structure in Pisha sandstone areas to different stumping.docx]

**Response of soil bacterial community structure in Pisha sandstone areas to different stumping**

Lu Cheng^1*^，Guihua Wang^1*^，Yuefeng Guo^1^，Zhiqiang Zhang^2^，Chengfu Zhang^1^，Min Han^1^，Yajie Feng^1^，Yang Song^1^

^1^ College of Desert Control Science and Engineering，Inner Mongolia Agricultural University，Hohhot，Inner Mongolia，China.

^2^ Old Barag County Forestry and Grassland Bureau Comprehensive Support Center, Hulun Buir, Inner Mongolia, China

First Author：

Lu Cheng^1^，Guihua Wang^1^

^1^ College of Desert Control Science and Engineering，Inner Mongolia Agricultural University，Hohhot，Inner Mongolia，China

Corresponding Author:

Yuefeng Guo^1^

^1^ College of Desert Control Science and Engineering，Inner Mongolia Agricultural University，Hohhot，Inner Mongolia，China.

Email address: guoyuefeng0525@126.com

**Abstract**

**Background.**The Pisha sandstone region of Inner Mongolia is one of the areas most severely affected by soil erosion in the world. Stumping is a widely used practice for rejuvenating degraded sea buckthorn (*Hippophae rhamnoides*) plantations, but how stumping depth affects soil bacterial communities and the underlying drivers remains unclear. We hypothesized that (1) 15 cm stumping depth would maximize soil nutrient availability and bacterial diversity, and (2) shifts in bacterial community composition would be primarily driven by soil nutrients and physical properties.

**Methods.**We conducted a field experiment in a 10-year-old declining sea buckthorn plantation, applying four stumping heights (0 cm, 10 cm, 15 cm, 20 cm) with uncut forest as control. Soil samples were collected from three compartments (root surface, rhizosphere, non-rhizosphere). Bacterial community composition was characterized by high-throughput sequencing of the 16S rRNA gene V4 region using primers 515F/806R with high-fidelity DNA polymerase. Soil physicochemical properties were measured using standard methods.

**Results.**Stumping at 15 cm height consistently exhibited the strongest effects across all soil compartments. Compared to other treatments and the control, this treatment significantly increased soil organic carbon, available nitrogen, phosphorus, and potassium, and enhanced bacterial α-diversity (Chao1, Shannon). At the family level, 15 cm stumping most strongly enriched *Oxalobacteraceae*, with relative abundance peaking at 38.9% in rhizosphere soil. Bacterial community composition shifted with stumping depth; however, β-diversity variation was primarily explained by spatial heterogeneity among the three plots( R² = 11.89%, P = 0.008), rather than stumping treatment itself (R² = 9.89%, P = 0.226). Redundancy analysis further revealed that *Actinobacteriota* abundance was positively correlated with soil organic matter, available nitrogen, available phosphorus, available potassium, and soil water content, but negatively correlated with soil bulk density.Membership function analysis confirmed that 15 cm stumping yielded the highest comprehensive scores across all soil compartments, particularly in root surface soil (0.54, 0.53, and 0.52 for root surface, rhizosphere, and non-rhizosphere soils, respectively).Taken together, these findings demonstrate that 15 cm stumping effectively improves soil nutrient conditions, enriches beneficial bacterial taxa, and reshapes microbial communities in degraded sea buckthorn plantations, with the strongest effects occurring in root-associated soils.

**Key words**: Pisha sandstone areas；Hippophae rhamnoides；Illumina MiSeq；Bacterial community diversity

# Introduction

**Abstract Guidance – remove this box before submitting!**

- No more than approx. 500 words (or 3,000 characters).
- Self-contained and concisely describe the reason for the work, methodology, results, and conclusions. Uncommon abbreviations should be spelled out at first use. Do not include footnotes or references.
- Headings in structured abstracts should be bold and followed by a period. Each heading should begin a new paragraph. For example:

**Background.** The background section text goes here. Next line for new section.

**Methods.** Methods section here, then new line.

**Results.** Results section here, then new line.

**Discussion.** Discussion section here.

The Pisha sandstone area of Inner Mongolia is one of the regions most severely affected by soil erosion in the world^[1]^. Due to the unique properties of Pisha sandstone, which turns into mud when exposed to water and sand when exposed to wind, the ecological environment of this region is fragile. The area is a major source of coarse sand for the Yellow River’s sediment^[2]^, which not only leads to significant sediment deposition in the river’s lower reaches but also triggers a series of serious ecological and environmental issues^[3-5]^. Sea buckthorn (Hippophae rhamnoides) is a shrub or small tree characterized by a well-developed root system, strong tillering ability, and rapid reproduction. In the Pisha sandstone area of Inner Mongolia, sea buckthorn is valued for its unique ecological benefits^[6]^. Additionally, sea buckthorn demonstrates strong soil adaptability and environmental resilience^[7,8]^. However, in recent years, environmental factors such as scarce rainfall and the region’s arid climate have caused the decline or even death of large areas of sea buckthorn forests. The practice of stumping—cutting back the above-ground branches of shrubs or semi-shrubs to stimulate new growth from the rootstock—has been shown to significantly promote the growth of suckers in the above-ground parts of sea buckthorn stands and enhance the vitality of the root system^[6]^. Soil harbors microbial communities with a high degree of diversity, which may contribute to plant growth, nutrient uptake, disease resistance, and stress tolerancey^[9]^.The microbiota in soil (non-inter-root, inter-root, and root surface) is directly or indirectly influenced by biotic and abiotic factors, such as climatic conditions and soil physico-chemical properties^[10]^. Plants and microorganisms in the inter-root soil engage in reciprocal feedforward regulation and are closely linked organisms^[11]^.

The development of modern molecular biotechnology is advancing rapidly, and many researchers—both domestically and internationally—are focusing on the study of soil microorganisms, which have become an important indicator for evaluating ecological environment quality ^[12]^. Recent studies indicate that different reclamation methods significantly affect soil microbial populations ^[13,14]^ Investigations into soil microbial communities in reclaimed mining areas across diverse regions have revealed both positive and negative correlations between soil salinity and microbial community structure and abundance ^[15-17]^. Researchers have used the BIOLOG-ECO microplate assay and dilution plate method to assess soil microbial community structure ^[18-20]^. However, due to methodological limitations, these studies cannot fully reflect the structural and quantitative characteristics of soil microbial communities ^[21,22]^. Currently, fewer studies have examined the response of soil microbial (non-rhizosphere, rhizosphere, and root-surface) community structure to different stumping treatments of sea buckthorn.To address this gap, we hypothesized that: (1) 15 cm stumping would maximize soil nutrient availability and bacterial α-diversity; (2) the abundance of beneficial taxa (e.g., *Oxalobacteraceae*) would peak under 15 cm stumping; (3) shifts in bacterial community composition would be primarily driven by soil nutrient-related parameters rather than physical properties; and (4) stumping treatment effects on overall community structure would be detectable but secondary to spatial heterogeneity.To test these hypotheses, we conducted a field experiment examining soil bacterial communities (non-rhizosphere, rhizosphere, and root-surface) and physicochemical properties under four stumping heights (0, 10, 15, and 20 cm) in a degraded sea buckthorn plantation in the Pisha sandstone region.

# Materials & Methods

**Study Area.** The study area is located in the Pisha Sandstone Soil and Water Conservation Science and Technology Demonstration Area in Nuanshui Township, Jungar Banner, Ordos City, Inner Mongolia. The geographic coordinates are 39°42′ to 39°50′ N latitude and 110°25′ to 110°48′ E longitude. This area belongs to a temperate continental monsoon climate, characterized by long, cold winters, short hot summers, and significant temperature fluctuations in spring and autumn. The average annual temperature ranges from 5.3°C to 7.6°C. The average frost-free period is 150 days, with an average of 2,981 hours of sunshine annually. The average annual precipitation is 400 mm, with the extreme maximum annual rainfall reaching 636.5 mm and the extreme minimum at 143.5 mm. Most of the rainfall occurs in August each year^[23]^. The primary soil types in the region include loess, chestnut calcareous soil, and wind-sand soil. After native vegetation was severely damaged, the area now largely relies on artificial vegetation cover. The natural vegetation consists mainly of species such as dogwood (*Setaria viridis*), flower stick (*Hedysarum scoparium*), hyacinth (*Achnatherum splendens*), and tamarisk (*Tamarix chinensis*), while artificial vegetation mainly consists of sea buckthorn and mountain almond (*Armeniaca sibirica*). The artificial vegetation is dominated by sea buckthorn, mountain apricot (*Armeniaca sibirica*), and lemon mallow (*Caragana korshinskii*), among others.

**Test Methods.** In this experiment, a 10-year-old sea buckthorn plantation forest with a plant spacing of 2 m × 4 m was selected as the research subject, and stumping treatments were applied. Following the principle of maintaining consistency in standing and growth conditions, treatment plots were set up with stumping heights of 0 cm (A), 10 cm (B), 15 cm (C), and 20 cm (D), along with a control group without stumping (E). Each sample plot covered an area of 50 m × 50 m, with three replicate sample plots for each treatment^[23]^. In September 2022, soil sampling was conducted. Samples were collected from root surface soil (S), inter-root soil (W), and non-root soil (R). In each sample plot, three healthy sea buckthorn plants with similar plant height and basal diameter were selected for stumping. Surface litter was removed, and soil samples were taken from a depth of 0 to 10 cm, using the ground surface as the reference point. Excess soil around the root system was shaken off manually, leaving about 1 mm of soil behind. The root system was then cut into small segments of approximately 5 cm using sterile gloves and sterile scissors, and the samples were packed into 50 mL test tubes and stored in liquid nitrogen. These root samples were later transferred to the laboratory and separated into inter-root and root surface soil, following the procedures described by Edwards^[24]^ et al. and Durán^[25]^ et al. The collected soil samples were individually placed into sterile soil sample dispensing tubes and stored in a freezer at -70°C for 16S rDNA sequencing.

**Analysis of Soil Physical and Chemical Properties.** The methods for analyzing the soil’s physicochemical properties are shown in Table 1^[23]^:

**Soil Microbial Diversity Profiling.** The structural composition and diversity of the soil bacterial community were determined by Lianchuan Bioinformatics Co.

Soil samples were collected following strict sterile procedures to avoid contamination by exogenous microorganisms. Genomic DNA was extracted using the CTAB method. The purity and concentration of the DNA were assessed by agarose gel electrophoresis (conditions: 1% gel concentration, 100 V, 40 minutes).

Qualified DNA samples were diluted with sterile water to a concentration of 1 ng/μL to serve as templates. The V4 region of the bacterial 16S rRNA gene was amplified using primers 515F (5‘-GTGYCAGCMGCCGCGGTAA-3’) and 806R (5’-GGACTACNVGGGTWTCTAAT-3‘) with a high-fidelity DNA polymerase^[24]^. The PCR products were checked on a 2% agarose gel, then pooled in equimolar amounts. Target bands were excised and purified using a gel extraction kit. Sequencing libraries were constructed using the TruSeq® DNA PCR-Free Sample Preparation Kit. After quantification with Qubit and validation by Q-PCR, the qualified libraries were sequenced on an Illumina HiSeq 2500 platform (PE250) by LC-Bio Technology Co., Ltd^[24-26]^.

The raw sequencing data were processed using the following bioinformatics pipeline:

Demultiplexing and Initial QC: Samples were demultiplexed based on barcodes. Primer and adapter sequences were trimmed using Cutadapt (v1.9). Paired-end reads were merged using FLASH (v1.2.8) based on their overlap.

Quality Filtering: Sequences were quality-filtered using fqtrim with a sliding window of 100 bp. The 3' end of a read was truncated when the average quality score within the window fell below 20. Reads shorter than 100 bp after truncation or containing more than 5% ambiguous bases (N) were discarded.

Denoising and ASV Inference: Denoising, chimera removal, and Amplicon Sequence Variant (ASV) calling were performed using the DADA2 plugin within QIIME2. Singleton ASVs (features appearing only once across all samples) were removed by default.

Core ASV Table Construction: To reduce noise from low-abundance sequences, the resulting raw ASV table was filtered. In this study, a core feature table containing 24,501 ASVs was generated and used for all downstream analyses.

Taxonomic Annotation: Representative ASV sequences were taxonomically classified using the feature-classifier classify-sklearn command in QIIME2, against the SILVA 138 reference database, with a confidence threshold set at 0.7.

Data Normalization: To mitigate the effect of uneven sequencing depth on ecological diversity metrics, the ASV abundance table was rarefied (subsampled) to 40,000 sequences per sample using the feature-table rarefy function in QIIME2. This depth was lower than the original sequencing depth of all samples in this dataset (range: 38,573–70,800), ensuring 100% sample retention.

Diversity Analysis: Alpha Diversity: Based on the rarefied data, indices including Shannon, Simpson, Chao1, Good's coverage, and Pielou’s evenness (Pielou_e) were calculated. Beta Diversity: Beta diversity was assessed based on Bray-Curtis dissimilarity matrices. Principal Coordinate Analysis (PCoA) was performed for visualization, and permutational multivariate analysis of variance (PERMANOVA) was used to test for significant differences between groups.

Differential Species Analysis: Differential species analysis between groups was performed based on normalized relative abundance data. Non-parametric tests were selected according to the experimental design: Fisher's exact test for comparisons without biological replicates; the Mann-Whitney U test for comparing two groups with replicates; and the Kruskal-Wallis test for multi-group comparisons with replicates. The significance level was set at p < 0.05. Prior to downstream analysis, all sequences classified as chloroplast or mitochondrial were removed.

**Data Processing.** Data were collated using Excel, with one-way ANOVA performed using SPSS 26. Graphing was conducted with Origin 2021, and redundancy analysis (RDA) was performed using Canoco 4.5 software. Data are presented as the mean ± standard deviation. Prior to conducting one-way analysis of variance (ANOVA), the underlying assumptions were verified. Specifically, the Shapiro-Wilk test was used to assess normality, and Levene's test was used to assess the homogeneity of variances. The results confirmed that the data in all groups met the assumptions of normality (all p > 0.05) and homogeneity of variances (p > 0.05), thus justifying the use of parametric ANOVA. When ANOVA indicated a significant effect, Tukey's post-hoc test was applied for pairwise comparisons.

To visualize the overall differences in microbial community structure between samples, Principal Coordinates Analysis (PCoA) was first performed based on the Bray-Curtis distance matrix. Specifically, PCoA was conducted using the ‘cmdscale’ function within the vegan package (version 2.7-2) of R software (version 4.5.2). The coordinates of the first two principal coordinate axes (PCoA1 and PCoA2) were extracted, along with the percentage of total variation explained across samples. Subsequently, scatter plots were generated using the ‘ggplot2’ package (version 4.0.1), with PCoA1 and PCoA2 as axes. Sample points were colored according to different experimental treatment groups to visually demonstrate the clustering of samples within each treatment group and the separation trends between groups. The significance level was set at p < 0.05 for all tests.

The comprehensive evaluation was based on the affiliation function method.

The formula is：

If the indicator is positively correlated with plant growth,

$X\left( U \right)=\frac{X-X_{\min}}{X_{\max}-X_{\min}}$	（1）

If the indicator is negatively correlated with plant growth, the

$X\left( U \right)=1-\frac{X-X_{\min}}{X_{\max}-X_{\min}}$	（2）

Where X represents the measured value of the indicator, Xmax is the maximum value of the measurement and Xmin is the minimum value of the measurement.

# Results

**Effects of Different Stumping Treatments on the Physico-Chemical Properties of Sea Buckthorn Soil.** The physicochemical properties of sea buckthorn soil following different stumping treatments are summarized in Table 2. Organic matter content was highest with the 15 cm and 10 cm stumping treatments, measuring (21.40 ± 0.95) g/kg and (20.08 ± 0.99) g/kg, respectively. No significant difference was found between these two treatments (p > 0.05), though both were significantly higher than the other treatments (p < 0.05). This indicates that both 15 cm and 10 cm stumping favor organic matter accumulation.

The maximum concentration of readily available nitrogen(AN) occurred at the 15 cm stumping, at (20.20 ± 1.09) mg/kg, significantly exceeding the other treatments. This demonstrates that the 15 cm stumping markedly enhances the readily AN content. The maximum concentration of readily available phosphorus(AP) was observed at 15 cm (8.056 ± 0.82 mg/kg), significantly exceeding other treatments (p < 0.01). This shows that 15 cm stumping most effectively enhances readily available phosphorus. The maximum concentration of readily available potassium(AK) was also observed at 15 cm stumping, at (64.348 ± 0.72) mg/kg, significantly higher than in other treatments (p < 0.05).

The highest soil moisture content(SMC), was recorded at the 10 cm stumping height, at (10.499 ± 0.27%), significantly higher than in all other treatments (p < 0.05). This indicates that a 10 cm stumping significantly increased SMC, with the 15 cm stumping also showing some improvement. The highest soil bulk density（BD） occurred in the control group (1.41 g/cm³), significantly higher than in the 15 cm and 20 cm stumping treatments (p < 0.05), but not significantly different from full stumping removal or the 10 cm stumping treatment (p > 0.05). This indicates that the control group exhibited the highest BD, while the 15 cm stumping group showed the lowest, suggesting a looser soil structure under the 15 cm stumping treatment.

In summary, the 15 cm stumping produced the most pronounced improvements in soil properties

**Analysis of the Number of ASVs in Samples under Different Stumping Treatments.** To demonstrate the number of shared versus unique Amplicon Sequence Variants (ASVs) between individual samples or subgroups, we visualized this using a Venn diagram. In the Venn diagram shown in Figure 1, each circle represents a stumping treatment, and the overlap between the circles indicates the number of ASVs shared between different treatments, while the non-overlapping part shows the number of ASVs unique to each treatment.

For the R (root surface soil), a total of 6,693 ASVs were identified across the five stumping treatments, with 88 ASVs shared between treatments. The number of unique ASVs was 639, 387, 368, 844, and 1,807 for each treatment, respectively. For the W (rhizosphere soil), a total of 9,180 ASVs were identified, with 53 co-owned ASVs, and unique ASVs numbered 1,525, 1,149, 250, 2,161, and 929, respectively. For the S (non-rhizosphere soil), 10,872 ASVs were identified, with 168 co-owned ASVs and 1,107, 1,442, 1,335, 477, and 1,695 unique ASVs, respectively.

The percentage of shared ASVs was as follows: R: 1.31%, W: 0.58%, S: 1.55%.

The percentage of unique ASVs was as follows: R-specific ASVs: 9.55%, 5.77%, 5.50%, 12.61%, and 27.00%, W-specific ASVs: 16.61%, 16.61%, 16.52%, 5.10%, 12.52%, 5.10%, 23.54%, and 10.12%. S-specific ASVs: 10.18%, 13.26%, 12.28%, 4.39%, and 15.59%

The bacterial ASV ratios for the unlevelled and fully levelled stumping soils were approximately: R: 0.43, W: 1.64, S: 0.74. 10 cm stumping: R: 0.75, W: 0.82, S: 1.96. 15 cm stumping: R: 1.45, W: 5.33, S: 0.90. 20 cm stumping: R: 1.40, W: 1.20, S: 0.79.

The results indicate that 10 cm stumping primarily affected bacterial ASVs in the root surface soil, while 15 cm stumping had a more significant effect on species composition at the ASV level in both the non-root surface soil and root surface soil.

**Analysis of Soil Bacterial Community Structure α-diversity under Different Stumping Treatments.** According to the results of the α-diversity index, as shown in Table 3, Good's coverage values were 1.00 for all samples, indicating that the sequencing depth captured the full bacterial diversity. The Chao1 index, which estimates the total number of species in a community, including those potentially not observed, was also used to assess species richness. In the case of R, the highest number of species was observed in the 15 cm stumping treatment, followed by the 10 cm stumping treatment; for W, the highest species richness was found in the 15 cm stumping treatment, followed by the 0 cm stumping treatment; and for S, the highest species richness occurred in the 15 cm stumping treatment, followed by the 10 cm stumping treatment.

The Shannon index measures diversity, with higher values indicating higher diversity. The Shannon index was highest for R, W and S in the 15 cm stumping treatment, suggesting that this treatment enhances microbial diversity.

The Simpson index ranges from 0 to 1, with a value of 0 indicating the lowest diversity (only one species in the community) and a value of 1 indicating the highest diversity (extremely rich species with an even distribution). In this study, the Simpson index for most of the samples ranged between 0.8 and 1.0, indicating a high degree of evenness in the distribution of species.

Pielou’s index, which measures the evenness of species distribution (the higher the value, the more uniform the distribution), showed that S had the highest evenness compared to R and W, indicating that S had the highest Pielou index in the 15 cm stumping treatment.

## **Analysis of Soil Bacterial Community Structure β-Diversity under Different Stumping Treatments.** To analyze the effects of different treatments and spatial location (the three plots R, S, and W) on microbial community structure, we conducted a multivariate PERMANOVA analysis based on the Bray-Curtis distance matrix (permutations = 999). The results indicated that the overall model incorporating both block and treatment factors was significant (Pseudo-F = 1.76, P = 0.014). Further decomposition of factor effects revealed that differences among the three plots (R, S, and W) had a highly significant influence on community variation (R² = 11.89%, P = 0.008), indicating substantial differences in community composition between the R, S, and W blocks. In contrast, after controlling for these plot-to-plot differences, differences between treatments did not reach statistical significance (R² = 9.89%, P = 0.226). Additionally, the PERMDISP test for homogeneity of dispersion between groups was non-significant (P = 0.833), confirming the reliability of the PERMANOVA results and ruling out variation within groups as an explanatory factor.

Visualization based on Principal Coordinate Analysis (PCoA) (Figure 2) corroborated these statistical findings. The PCoA1 and PCoA2 axes explained 43.44% and 6.66% of the community variation, respectively. The plot reveals that sample points first form distinct clusters based on plot block (represented by different shapes). For instance, all samples from Plot Block R tend to cluster in a specific region, visually demonstrating that the plot block effect is the primary driver of community variation. Simultaneously, sample points from different treatments (denoted by distinct colors) exhibit spatially intermingled distributions without forming independent clusters. This aligns with the statistically insignificant treatment effects. Post-hoc pairwise comparisons also failed to identify any significant differences between treatment groups (all corrected P > 0.05).

**Analysis of the Bacterial Community Structure of Sea Buckthorn Soil under Different Stumping Treatments.** Soil bacterial communities exhibited high diversity across all stumping treatments, as indicated by the Shannon diversity index (ranging from 5.69 to 9.80) and Chao1 richness index (ranging from 791.77 to 2432.13) (Table 3). The 15 cm stumping treatment consistently showed the highest values for both indices across all soil compartments. At the phylum level (Figure 3), the top ten dominant bacterial phyla in terms of relative abundance were *Proteobacteria*, *Actinobacteriota*, *Acidobacteriota*, *Firmicutes*, *Chloroflexi*, *Bacteroidota*, *Cyanobacteria*, *Globacteria*, and *Bacteriophage*, including *Bacteroidota*, *Cyanobacteria*, *Planctomycetota*, *Gemmatimonadota*, and *Myxococcota*. The relative abundance of *Proteobacteria* was the highest across all treatments and soils, with the highest relative abundance observed in R and W in the 15 cm stumping treatment. The proportions of *Proteobacteria* and *Actinomycetes* were higher in S, showing minimal variation across different stumping treatments.

At the family level, all treatments exhibited highly consistent community structure characteristics (Figure 4). *Oxalobacteraceae*, *Pseudomonadaceae*, and *Hafniaceae* stably dominated across the three regions, forming the core microbiota. Among these, *Oxalobacteraceae* had the highest relative abundance, generally ranging between 20% and 40%. Further comparisons revealed reproducible microbial enrichment patterns for the same treatment across regions. For example, the 15 cm stumping treatment (including RC, SC, and WC) most significantly enriched *Oxalobacteraceae*, with its abundance peaking at 38.9% in the WC treatment. In contrast, complete stumping (including RA, SA, and WA) generally resulted in lower abundances of dominant families, such as *Oxalobacteraceae*, which only reached 15.2% in the WA treatment.

Notably, data from the S region exhibited relatively high within-group variation. For example, the abundance of *Oxalobacteraceae* in the SB treatment ranged from 4.1% to 40.7%, reflecting the inherent spatial heterogeneity of soil microorganisms in the field. Nevertheless, the consistent treatment effects and stable core microbiota composition indicate that stumping is a key driver shaping soil bacterial community structure.

**Relationship between Bacterial Dominant Phylum and Soil Physico-Chemical Properties of Sea Buckthorn Soil under Different Stumping Treatments.** To investigate the influence of environmental factors and soil bacterial communities, we conducted an RDA (Redundancy Analysis) of soil physicochemical properties and bacterial community composition at the phylum level. The results of this analysis are shown in Figure 5. The AP, AK, and BD rays were longer at the phylum level in S, indicating that these factors had a greater influence on the structure of the bacterial community at the phylum level. Similarly, AP and AN showed longer vectors in the W ordination, suggesting that they explained more variation in bacterial community composition compared to other factors.

In S, W, and R, AN, SOM, AP, AK, and w were positively correlated with the *Actinobacteriota* phylum, while BD was negatively correlated with it. These results suggest that soil microorganisms and soil physicochemical properties under different stumping treatments have a mutual influence on each other.

**Comprehensive Analysis of Sea Buckthorn Characteristics under Different Stumping Treatments.** In this study, we comprehensively analyzed and evaluated the indices of sea buckthorn soil and microorganisms under different stumping treatments using the affiliation function method. The indicators of sea buckthorn, namely: soil organic matter (X1), quick-acting phosphorus (X2), quick-acting nitrogen (X3), quick-acting potassium (X4), soil water content (X5), soil bulk density (X6), number of ASVs (X7), Shannon index (X8), Simpson index (X9), Chao1 index (X10), and Pielou's evenness (X11), were used for comprehensive evaluation.

As shown in Tables 4-6, the affiliation function values for the indicators of soil microorganisms in sea buckthorn under different stumping leveling modes were highest in the 15 cm stumping leveling for root surface soil, inter-root soil, and non-inter-root soil.

**Discussion**

Soil physicochemical properties are fundamental drivers of bacterial community structure ^[27–29]^. Our redundancy analysis (RDA) revealed that AN, SOM, AP, and AK were the primary edaphic factors shaping bacterial communities under different stumping treatments. These nutrient-related parameters showed consistent positive correlations with most dominant phyla, particularly with *Actinobacteriota*, which exhibited strong positive associations with SOM, AN, AP, AK, and SWC, and a negative correlation with BD. This aligns with Lin et al. ^[30]^ and Zhao et al. ^[31]^, who reported similar linkages between microbial composition and soil nutrient status. The marked influence of nutrient availability suggests that stumping primarily affects microbial communities through resource-mediated pathways, rather than via direct physical disturbance.

Among all treatments, 15 cm stumping consistently produced the strongest improvements in soil organic carbon, available nutrients, and bacterial α-diversity (Chao1, Shannon). This pattern likely reflects an optimal resource balance: 15 cm stumping stimulates root exudation and fine root turnover without causing excessive carbon limitation or water stress, thereby enhancing both nutrient pools and microbial richness. In contrast, 20 cm stumping or complete stumping removal may reduce plant carbon input, while insufficient stumping (0 or 10 cm) fails to sufficiently rejuvenate root activity ^[32–34]^. These findings support our hypothesis that moderate disturbance optimizes soil–microbe feedbacks in degraded plantations.

At the family level, *Oxalobacteraceae*, *Pseudomonadaceae*, and *Hafniaceae* formed a stable core microbiota across all soil compartments. Notably, 15 cm stumping most strongly enriched *Oxalobacteraceae*, with relative abundance peaking at 38.9% in rhizosphere soil. Members of *Oxalobacteraceae* are known copiotrophs that respond rapidly to increased nutrient availability and are often associated with plant growth promotion and suppression of soil-borne pathogens ^[35, 36]^. Their consistent enrichment under 15 cm stumping suggests that this practice not only improves soil fertility but also enhances the functional potential of the rhizosphere microbiome. The high within-group variation observed in non-rhizosphere soil (e.g., *Oxalobacteraceae* abundance ranging from 4.1% to 40.7% in the SB treatment) reflects the inherent spatial heterogeneity of field soils, yet the reproducible treatment effects underscore the ecological relevance of stumping as a management tool.

Although stumping significantly altered individual taxa and α-diversity, β-diversity was primarily governed by spatial heterogeneity among plots rather than treatment itself. PERMANOVA confirmed that differences among plots explained a significant portion of community variation (P = 0.008), whereas treatment effects were non-significant after accounting for spatial structure (P = 0.226). This result is consistent with studies showing that pre-existing soil gradients often override the effects of short-term management interventions ^[37]^. The non-significant PERMDISP test (P = 0.833) further confirms that the lack of a treatment effect on community composition is genuine and not an artifact of dispersion heterogeneity. Thus, stumping effects on overall community structure are superimposed on, rather than replacing, the strong natural heterogeneity of the Pisha sandstone soils.

At the phylum level, *Proteobacteria*, *Actinobacteriota*, and *Acidobacteriota* dominated across all treatments, consistent with their cosmopolitan distribution in terrestrial ecosystems ^[38, 39]^. The predominance of *Proteobacteria*—a phylum often linked to carbon mineralization and plant growth promotion—supports the notion that stumping maintains or enhances soil functional capacity ^[39, 40]^. *Actinobacteriota* are known to participate in organic matter decomposition [40], and their positive correlation with multiple nutrient indicators further implies that this phylum plays a key role in nutrient cycling under improved soil conditions. Negassa et al. found that *Actinobacteriota* and *Proteobacteria* primarily inhabit macroporous spaces, while *Acidobacteriota* are more prevalent in small pore spaces; conversely, microbial metabolic activity can also modify soil pore structure ^[41]^. This bidirectional interaction has positive implications for improving soil structure in the Pisha sandstone region. Thus, the observed shifts in *Actinobacteriota* and *Proteobacteria* under 15 cm stumping may not only reflect improved nutrient availability but could also contribute to long-term improvements in soil physical structure, creating a positive feedback loop between soil properties and microbial communities.

Taken together, our results demonstrate that 15 cm stumping effectively enhances soil nutrient status, bacterial diversity, and the abundance of beneficial taxa, with the most pronounced effects occurring in root-associated soils. However, these effects are context-dependent and modulated by inherent spatial variability. Future studies should integrate metagenomic or metatranscriptomic approaches to resolve the functional gene responses underpinning the observed taxonomic shifts ^[36]^, and long-term field trials are needed to assess the sustainability of stumping-mediated soil improvements. Such knowledge will aid in refining stumping regimes for soil conservation and plantation restoration in the Pisha sandstone region and analogous eroded landscapes.

**Conclusions**

This study investigated the effects of different stumping heights on soil physicochemical properties and bacterial communities in a degraded sea buckthorn plantation in the Pisha sandstone region. Our results demonstrate that 15 cm stumping consistently outperformed other treatments, significantly improving soil organic carbon, available nutrients, and bacterial α-diversity, while also enriching beneficial taxa such as Oxalobacteraceae (peaking at 38.9% in rhizosphere soil).

At the community level, β-diversity variation was primarily driven by spatial heterogeneity among experimental plots(R² = 11.89%, P = 0.008), with stumping treatment effects being detectable but secondary (R² = 9.89%, P = 0.226). Redundancy analysis further revealed that *Actinobacteriota* abundance was positively correlated with nutrient-related soil parameters (organic matter, available N, P, K, and moisture) and negatively correlated with BD, suggesting that stumping influences microbial communities mainly through resource-mediated pathways.

Membership function analysis confirmed that 15 cm stumping yielded the highest comprehensive scores across all soil compartments, particularly in root-associated soils. Collectively, these findings provide a microbial perspective for optimizing stumping regimes in soil erosion control and plantation restoration. Future studies should investigate the functional gene responses to stumping and explore the long-term dynamics of key microbial taxa under different management practices.

**References**

[1]Ambuque, Zhang Changshou. Effects of Clear-Cutting on Shrub Growth in PIsha-Sandstone Areas [J]. Inner Mongolia Forestry, 2013, (10): 10-11.

[2]GUO Yuefeng, BU Fanjing, QI Wei, WANG Xin, XU Yajie, LIU Lu.Response of physiological characteristics of sea buckthorn to flat stumping in the PIsha sandstone area of Inner Mongolia[J].Journal of Yangzhou University(Agriculture and Life Science Edition),2021,42(02):129-134.

[3]WANG Lijiu,LI Changming,DONG Jingliang.Distribution and lithological characteristics of PIsha sandstone[J].People's Yellow River,2013,35(12):91-93+97.

[4]XING Qi-Xin,RAO Liang-Yi,WANG Zhi-Zhen, HU Jianru, XU Yeqin. Characteristics of soil organic matter and quick-acting potassium in different types of PIsha sandstone areas in Inner Mongolia[J].Journal of Soil and Water Conservation,2019,33(06):257-264+272.

[5]ZHANG Pan,YAO Wenyi,XIAO Peiqing,LIU Guobin, YANG Chunxia. Study on the interactive superposition effect of multi-dynamic erosion in the PIsha sandstone area of the Yellow River Basin[J].Journal of Water Resources,2022,53(01):109-116.

[6]GUO Yuefeng, QI Wei, YAO Yunfeng, WANG Xin.Effects of stumping height on physiological characteristics of sea buckthorn in PIsha sandstone area[J].Journal of Ecological Environment,2020,29(06):1116-1122.

[7]YANG Zhenqi, QIN Fucang, YU Xiaojie, HAN Jun, RENG Xiaotong, QIAN Qiuying. Construction of soil quality evaluation index system for plantation forest land in PIsha sandstone area based on minimum data set[J].Soil Bulletin,2019,50(05):1072-1078.

[8]LU Lina,ZHAO Yuxing,HU Lifang, LI Weixiang, SHI Lin, RENG Yuyan, HAN Yiliang, HE Xiaohui. Effects of sea buckthorn (Hippophae rhamnoides) planting on soil bulk density, porosity and water storage capacity in Ordos PIsha sandstone area[J].Desert China,2015,35(05):1171-1176.

[9]Fernando T Maestre, Manuel Delgado-Baquerizo, Thomas C Jeffries, David J Eldridge , Victoria Ochoa , Beatriz Gozalo, José Luis Quero, Miguel García-Gómez, Antonio Gallardo, Werner Ulrich, Matthew A Bowker, Tulio Arredondo, Claudia Barraza-Zepeda, Donaldo Bran, Adriana Florentino, Juan Gaitán, Julio R Gutiérrez, Elisabeth Huber-Sannwald, Mohammad Jankju, Rebecca L Mau, Maria Miriti, Kamal Naseri, Abelardo Ospina, Ilan Stavi, Deli Wang, Natasha N Woods, Xia Yuan, Eli Zaady, Brajesh K Singh. Increasing aridity reduces soil microbial diversity and abundance in global drylands.[J]. Proceedings of the National Academy of Sciences of the United States of America,2015,112(51).

[10]ZHONG Yangquanwei,Sorensen Patrick O.,ZHU Guangyu,JIA Xiaoyu,LIU Jin,SHANGGUAN Zhouping,WANG Ruiwu,YAN Weiming. Differential microbial assembly processes and co‐occurrence networks in the soil‐root continuum along an environmental gradient[J]. iMeta,2022,1(2).

[11]GENG Shijun, WANG Bo, LIU Kan, SHANG Haiyan, QUAN Junjiao, LU Xiaoping, SHEN Qirong. Effects of special microbial fertilisers on the inter-root microbiota of soils with different successive stumping barriers[J]. Jiangsu Agricultural Journal, 2012, 28 (04): 758-764.

[12]XIAO Li, ZHAO Junfeng, HUANG Yimei, MA Renxiang, FANG Ying, AN Shaoshan.Research on physicochemical properties and enzyme activities of soil under different vegetation types in Yongli open-pit coal mine discharge site[J].Research on Soil and Water Conservation,2016,23(04):89-93.

[13]LI Yang. Research on soil microbial community structure and its diversity in sea buckthorn artificial forest in coal mine reclamation area[D].Yan'an University,2020.

[14]SHANG Wenqin, ZHU Liping, SUN Qingye, YANG Linzhang.Microbiological changes in tailing waste land during natural ecological restoration[J].Ecological Environment,2008(02):713-717.

[15]MA Jianjun. Ecological restoration of surface coal mines in hilly and gully areas of the Loess Plateau and its ecological effects[D].Inner Mongolia Agricultural University,2007.

[16]XIN Yuanzheng. Characteristics of microbial communities and influencing factors in degraded wetlands in the Yellow River Delta[D].Ocean University of China, 2009.

[17]Baldwin D.S.,Rees G. N.,Mitchell A. M., Watson G. and Williams J.The short-term effects of salinization on anaerobic nutrient cycling and microbial community structure in sediment from a freshwater wetland.Wetlands,2006,26(2):455-464.

[18]YANG Chao,LV Dantong,JIANG Shenyi,LIN Hao,SUN Junqi,LI Kangjia,SUN Juan. Soil salinity regulation of soil microbial carbon metabolic function in the Yellow River Delta, China[J]. Science of the Total Environment,2021,790.

[19]LI Yuxin,ZHAO Zhong,CHEN Jinquan,LIU Entian.Study on soil microbial diversity in sea buckthorn forest[J].Journal of Northwest A&F University (Natural Science Edition),2010,38(08):67-74+82.

[20]LIU Zhao, WEI Tianxing, ZHU Qingke, CHEN Jue, ZHAO Yanmin.Characteristics of microorganisms, enzyme activities and nutrients in typical woodland soils in loess hill and gully areas[J].Soil,2016,48(04):705-713.

[21]ZHANG Aimei,HAN Xueying,ZHANG Shihu,SUN Kun,WU Juyan,YU Jie,GUO Baomin.Diversity of endophytic bacteria in rhizomes of Tibetan sea buckthorn at different altitudes in the alpine region of Gannan[J].Bioresources,2018,40(02):114-119.

[22]Zhang Mingming. Bacterial diversity and relationship with soil nutrients in soil and rhizoma of sea buckthorn woodland in Shaanxi[D].Northwest Agriculture and Forestry University,2019.

[23]QI Huijuan,GUO Yuefeng,YAO Yunfeng,QI Wei, BU Fanjing, WANG Haoyue. Response of inter-root soil microbial community of sea buckthorn to flat stumping in PIsha sandstone area[J]. Soil Bulletin, 2024, 55 (02): 437-447.

[24]J. Gregory Caporaso, Christian L. Lauber, William A. Walters, Donna Berg-Lyons, Catherine A. Lozupone, Peter J. Turnbaugh, Noah Fierer, Rob Knight. Global patterns of 16S rRNA diversity at a depth of millions of sequences per sample[J].Proceedings of the National Academy of Sciences of the United States of America,2011,108 Suppl 1(S1):4516-22.

[25]Bolger Anthony M, Lohse Marc, Usadel Bjoern. Trimmomatic: a flexible trimmer for Illumina sequence data.[J]. Bioinformatics, 2014, 30: 2114-20.

[26]Deepak Reyon, Shengdar Q Tsai, Cyd Khayter, Jennifer A Foden, Jeffry D Sander & J Keith Joung. FLASH assembly of TALENs for high-throughput genome editing[J]. Nature Biotechnol., 2012, 30: 460-5.

[27]LIU Jing, ZHAO Yan, ZHANG Qiaoming, XU Shaojun. Effects of different utilisation methods on soil microbial biomass and community structure characteristics in the loess hilly area of western Henan[J]. Journal of Grass Industry, 2016, 25(08): 36-47.

[28]WANG Yanfa, WEI Shiping, CUI Hongpeng, SU Xin, ZHU Youhai, LU Zhenquan, HU Fei, LI Laipeng, ZHANG Shuai, LIU Hui. Distribution and diversity of microorganisms in the vertical profile of soil in the permafrost region of the Tibetan Plateau[J]. Microbiology Bulletin, 2016, 43(09): 1902-1917.

[29]WANG Nan, PAN Xiaocheng, WANG Chuankuan, BAI Shangbin. Effects of simulated acid rain on soil fungal structure and its diversity in the transition zone of moso bamboo broadleaf forest[J]. Environmental Science, 2020, 41(05): 2476-2484.

[30]LIN Chunying, LI Xilai, ZHANG Yuxin, SUN Huafang, LI Chengyi, JIN Liqun, YANG Xinguang, LIU Kai. Response of soil microbial community structure to different degradations in alpine swamp wetlands in the Yellow River source area[J]. Environmental Science, 2021, 42(08): 3971-3984.

[31]ZHAO Guohui, LI Zhezhe, HU Shaojun, CHENG Xiaofei, BOW Bi. Relationship between soil physical and chemical properties and microbial and plant communities in Yongcheng Lake wetland[J]. Journal of Northwest Forestry College, 2020, 35(05): 240-248.

[32]LI Haoxia, WEN Xuefei, TIAN Ying. Effects of growing season levelling stumping on the diversity of inter-plantation vegetation communities in lemonade forests in beamlands[J]. Ningxia Agriculture and Forestry Science and Technology, 2022, 63(02): 11-15+41.

[33]JIA Xiyang, ZHOU Jingjing, SU Tingting, ZHOU Yao, CHEN Jiabao, MA Hongbin, MA Jingli, WANG Xiaofang. Effects of flat stumping density on the habitat between artificial lemon groves in desert grassland[J]. Journal of Ecology, 2020, 40(12): 4126-4136.

[34]WANG Xia, HAI Lian, ZHAO Chen Guang, YANG Weichao, LIU Junliang, ZHU Yajuan. Effects of different stumping levelling methods on the growth of tamarisk[J]. Inner Mongolia Forestry Science and Technology, 2021, 47(03): 39-42+59.

[35]Spain AM, Krumhol LR, Elshahed M S. Abundance, composition, diversity and novelty of soil *Proteobacteria*[J]. Multidisciplinary Journal of Microbial Ecology, 2009, 3(8): 992-1000.[33]

[36]MEI Lan, HUANG Zaixing, SONG Zhaozhao, JIA Yulei, SU Dewei, LIN Hui, LIN Zhanshi. Effects of different growth periods of Giant Fungus Grass on soil physicochemical properties and bacterial community structure in an PIsha sandstone area[J]. Journal of Microbiology, 2019, 59(10): 2038-2050.

[37]ZHANG Hui, YU Duan, HANG Xiaoning, MA Lianjie, LIAO Dunxiu, LU Wencai. Effects of Different Nitrogen Application Treatments on Soil Microbial Diversity Under Rice-Vegetable Rotation[J]. Southwest China Journal of Agricultural Sciences, 2023, 36(03): 550-556.

[38]GUO Zhen, SHI Chendi. Prediction of bacterial community structure and function in soils with different compounding ratios[J]. Environmental Science and Technology, 2021, 44(01): 69-76.

[39]Vasvi Chaudhry, Ateequr Rehman, Aradhana Mishra, Puneet Singh Chauhan & Chandra Shekhar Nautiyal. Changes in bacterial community structure of agricultural land due to long-term organic and chemical amendments[J]. Microbial Ecology, 2012, 64(2): 450-460.

[40]Stefano Fazi, Stefano Amalfitano, Jakob Pernthaler, Alberto Puddu. Bacterial communities associated with benthic organic matter in headwater stream microhabitats[J]. Environmental Microbiology, 2005, 7(10): 1633-1640.

[41]ZHANG Zhiyong. Characteristics of soil microbial communities and soil quality evaluation of sea buckthorn forests in water and wind erosion interspersed areas[D]. Yan'an University, 2022.
