# Supplementary material for: Response of soil bacterial community structure in Pisha sandstone areas to different stumping
Source: PeerJ. 2026 May 4;14:e21234. doi: 10.7717/peerj.21234 (PMC13151931; doi:10.7717/peerj.21234)
Supplement: Supplemental Information 2 [file peerj-14-21234-s002.z01 › Data/DATA (3)/rda/Data152.pdf]

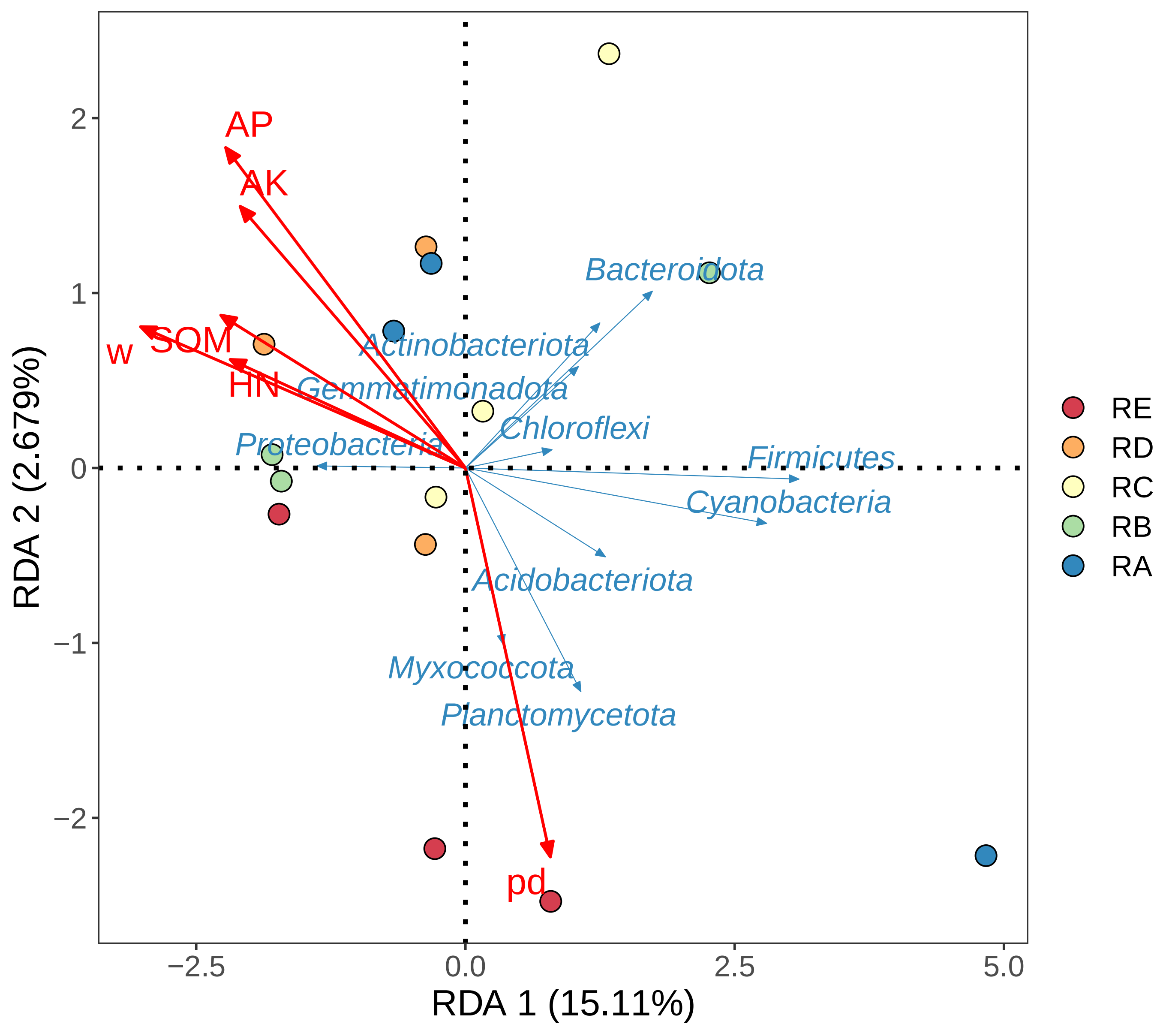
AP
AK
SOM
w
HN
Bacteroidota
Actinobacteriota
Gemmatimonadota
Chloroflexi
Proteobacteria
Firmicutes
Cyanobacteria
Acidobacteriota
Myxococcota
Planctomycetota
pd
RDA 2 (2.679%)
RDA 1 (15.11%)
2
1
0
−1
−2
−2.5
0.0
2.5
5.0
RE
RD
RC
RB
RA
