# Supplementary material for: Response of soil bacterial community structure in Pisha sandstone areas to different stumping
Source: PeerJ. 2026 May 4;14:e21234. doi: 10.7717/peerj.21234 (PMC13151931; doi:10.7717/peerj.21234)
Supplement: Supplemental Information 2 [file peerj-14-21234-s002.z01 › Data/DATA (3)/rda/Data154.pdf]

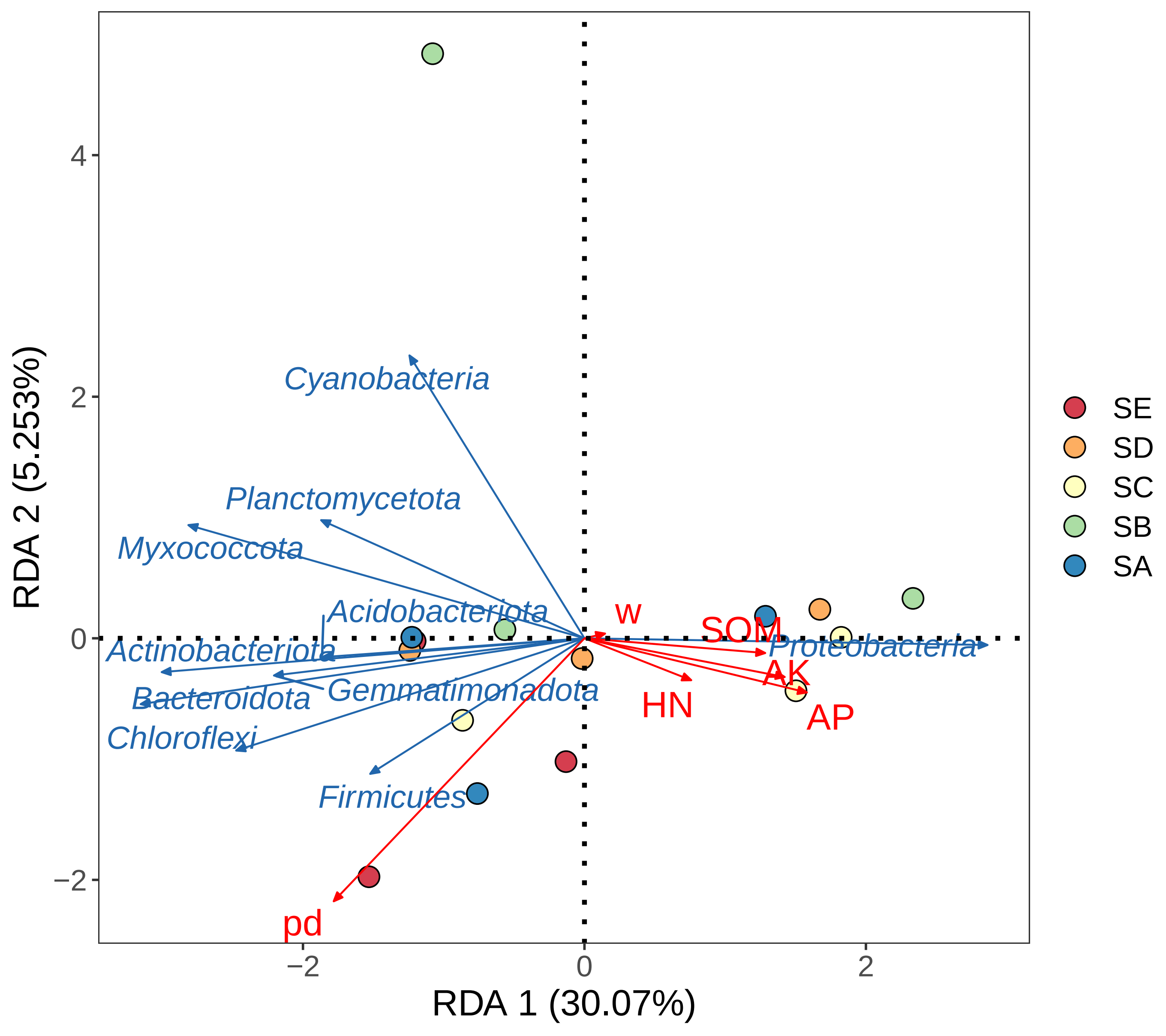

RDA 2 (5.253%)
RDA 1 (30.07%)
4
2
0
−2
−2
0
2
Cyanobacteria
Planctomycetota
Myxococcota
Acidobacteriota
Actinobacteriota
Gemmatimonadota
Bacteroidota
Chloroflexi
Firmicutes
Proteobacteria
w
SOM
AK
HN
AP
pd
SE
SD
SC
SB
SA
