# Supplementary material for: Response of soil bacterial community structure in Pisha sandstone areas to different stumping
Source: PeerJ. 2026 May 4;14:e21234. doi: 10.7717/peerj.21234 (PMC13151931; doi:10.7717/peerj.21234)
Supplement: Supplemental Information 2 [file peerj-14-21234-s002.z01 › Data/DATA (3)/rda/Data156.pdf]

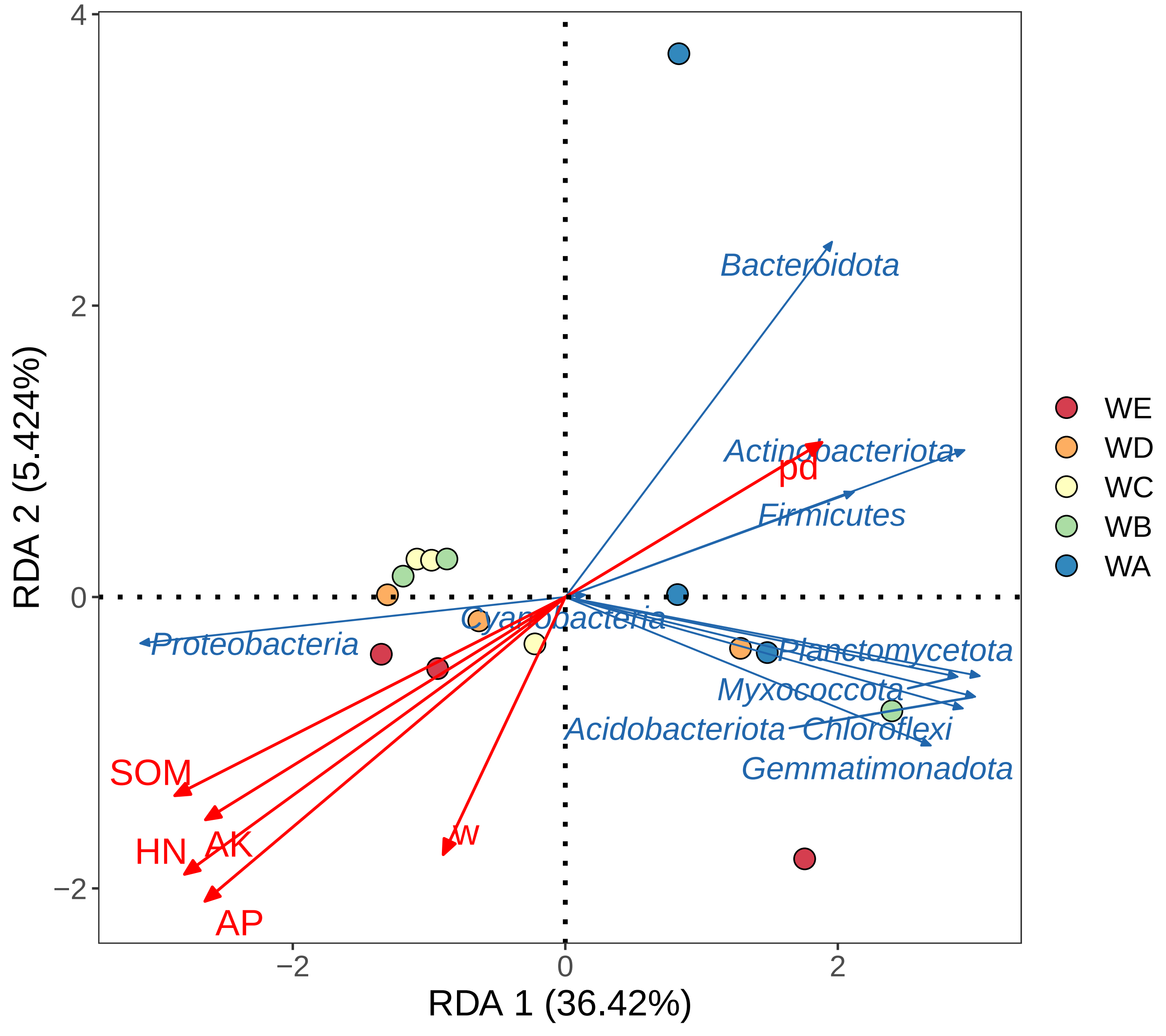

RDA 2 (5.424%)
RDA 1 (36.42%)
4
2
0
−2
−2
0
2
Bacteroidota
Actinobacteriota
pd
Firmicutes
Cyanobacteria
Proteobacteria
Planctomycetota
Myxococcota
Acidobacteriota
Chloroflexi
Gemmatimonadota
SOM
HN
AK
AP
w
WE
WD
WC
WB
WA
