# Supplementary material for: Response of soil bacterial community structure in Pisha sandstone areas to different stumping
Source: PeerJ. 2026 May 4;14:e21234. doi: 10.7717/peerj.21234 (PMC13151931; doi:10.7717/peerj.21234)
Supplement: Supplemental Information 2 [file peerj-14-21234-s002.z01 › Data/DATA (3)/rda/Data159.pdf]

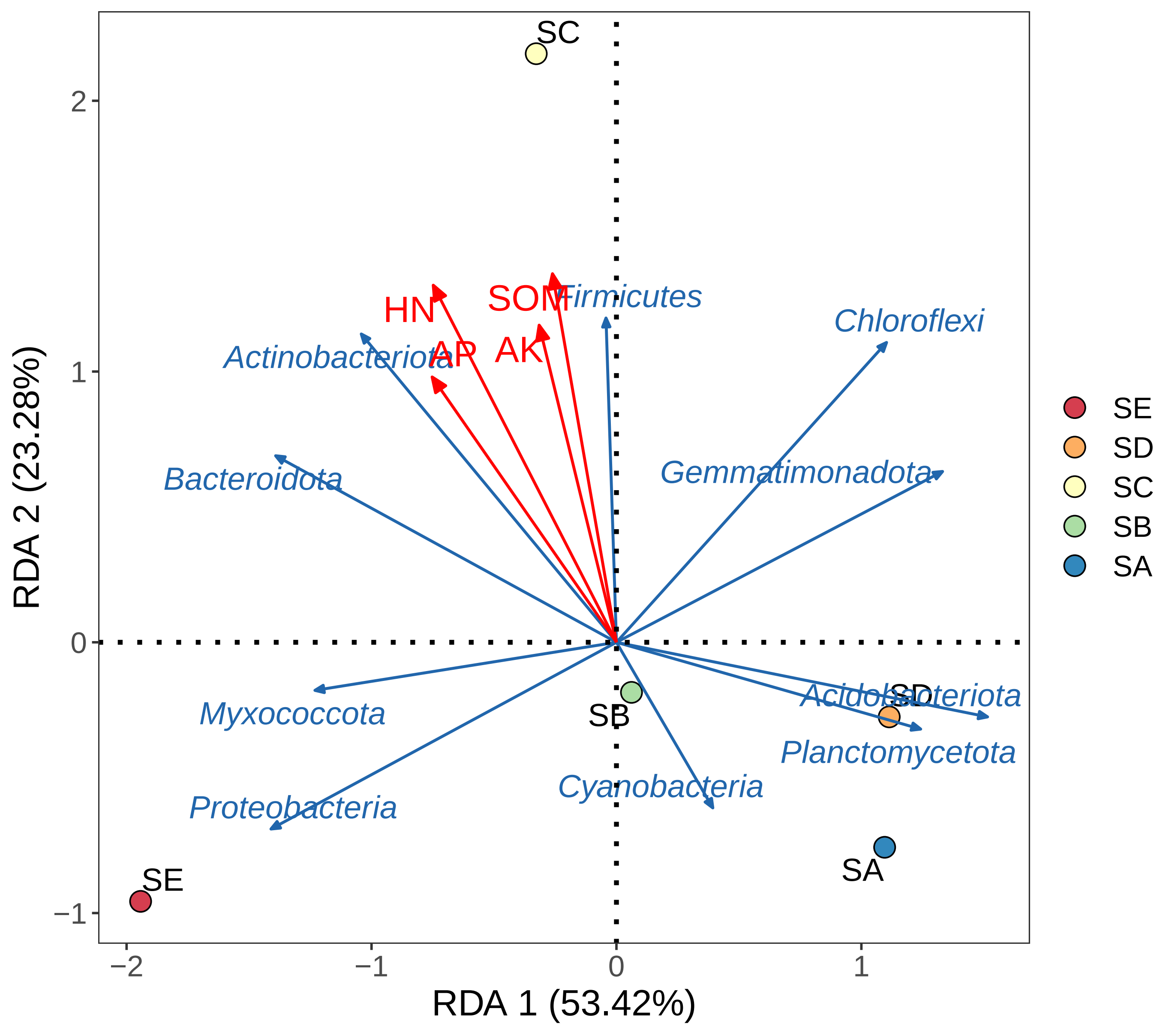
SC
SD
SB
SA
SE
HN
SOM
AP
AK
Firmicutes
Chloroflexi
Actinobacteriota
Bacteroidota
Gemmatimonadota
Acidobacteriota
Myxococcota
Planctomycetota
Cyanobacteria
Proteobacteria
RDA 2 (23.28%)
RDA 1 (53.42%)
2
1
0
−1
−2
−1
0
1
SE
SD
SC
SB
SA
