# Supplementary material for: Response of soil bacterial community structure in Pisha sandstone areas to different stumping
Source: PeerJ. 2026 May 4;14:e21234. doi: 10.7717/peerj.21234 (PMC13151931; doi:10.7717/peerj.21234)
Supplement: Supplemental Information 2 [file peerj-14-21234-s002.z01 › Data/DATA (3)/rda/Data161.pdf]

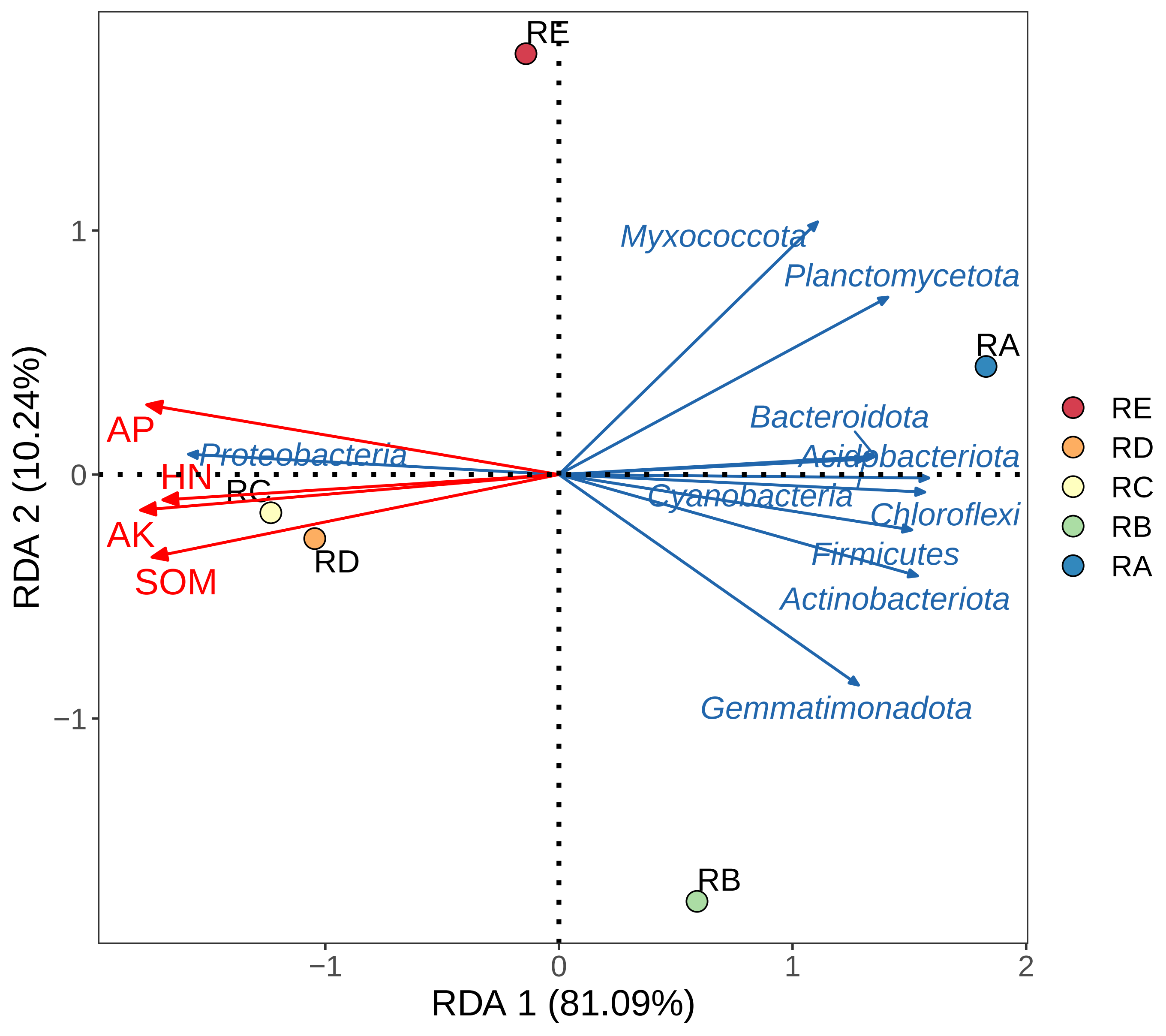

RE
RA
RB
RC
RD
AP
HN
AK
SOM
Myxococcota
Planctomycetota
Bacteroidota
Acidobacteriota
Proteobacteria
Cyanobacteria
Chloroflexi
Firmicutes
Actinobacteriota
Gemmatimonadota
1
0
−1
2
RDA 1 (81.09%)
RDA 2 (10.24%)
