# Supplementary material for: Response of soil bacterial community structure in Pisha sandstone areas to different stumping
Source: PeerJ. 2026 May 4;14:e21234. doi: 10.7717/peerj.21234 (PMC13151931; doi:10.7717/peerj.21234)
Supplement: Supplemental Information 2 [file peerj-14-21234-s002.z01 › Data/DATA (3)/rda/Data164.pdf]

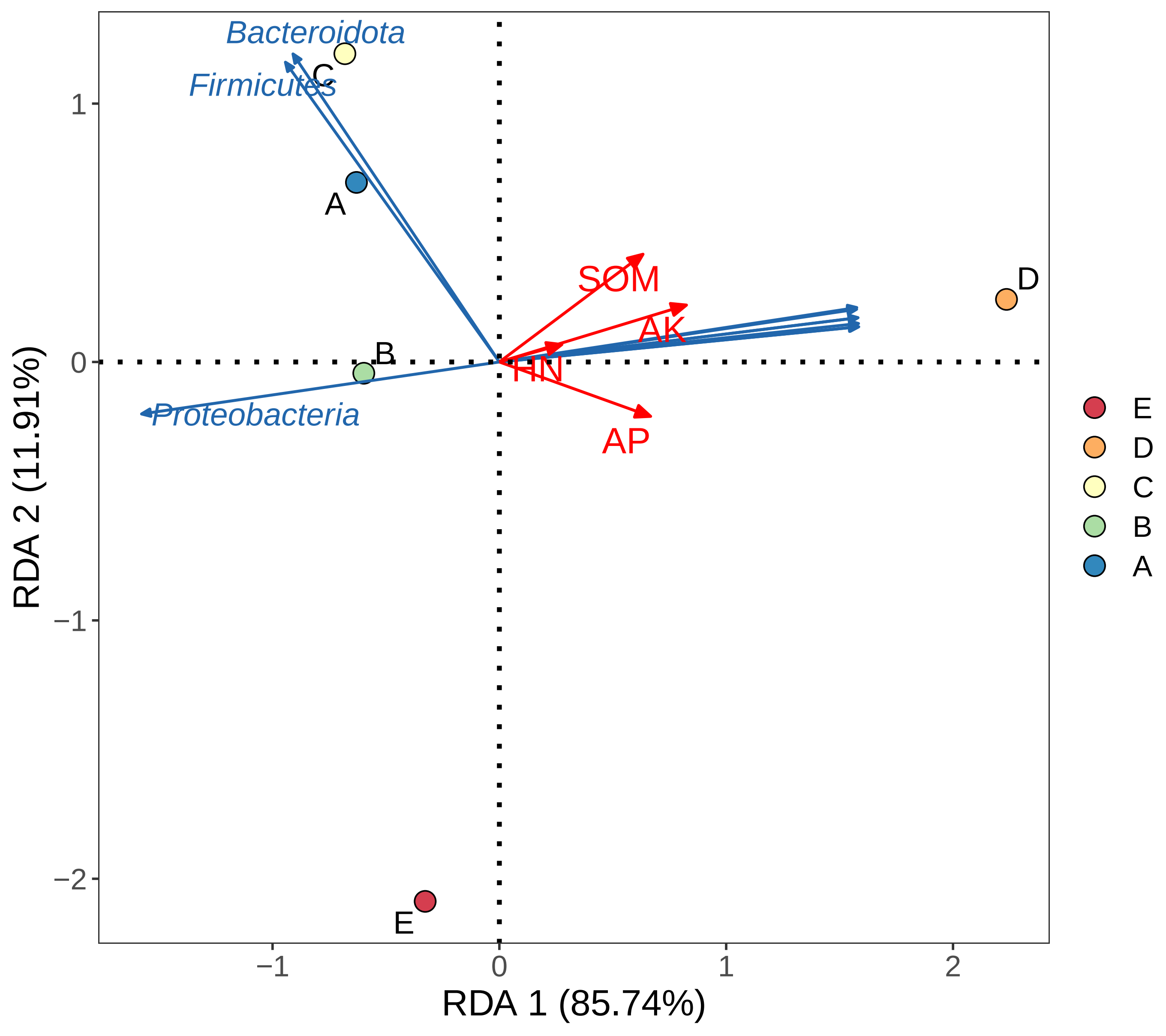
Bacteroidota
Firmicutes
Proteobacteria
SOM
AK
HN
AP
A
B
C
D
E
RDA 1 (85.74%)
RDA 2 (11.91%)
−1
0
1
2
−2
E
D
C
B
A
