# Supplementary material for: Response of soil bacterial community structure in Pisha sandstone areas to different stumping
Source: PeerJ. 2026 May 4;14:e21234. doi: 10.7717/peerj.21234 (PMC13151931; doi:10.7717/peerj.21234)
Supplement: Supplemental Information 2 [file peerj-14-21234-s002.z01 › Data/DATA (3)/rda/Data168.pdf]

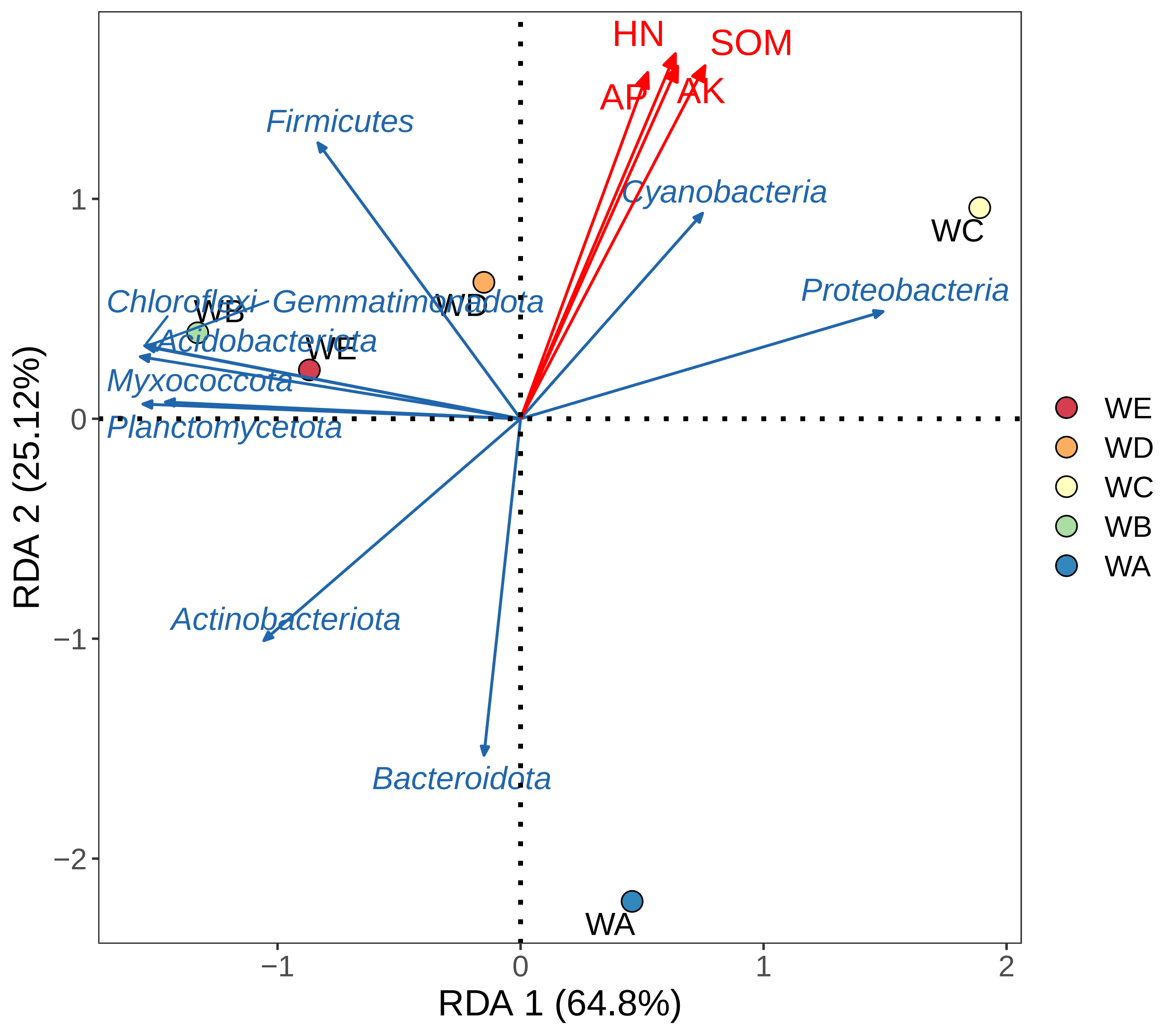
HN
SOM
AP
AK
Firmicutes
Cyanobacteria
WC
Proteobacteria
Chloroflexi
Gemmatimonadota
WB
WD
Acidobacteriota
WE
Myxococcota
Planctomycetota
Actinobacteriota
Bacteroidota
WA
1
0
−1
−2
−1
0
1
2
RDA 2 (25.12%)
RDA 1 (64.8%)
WE
WD
WC
WB
WA
