# Supplementary material for: Response of soil bacterial community structure in Pisha sandstone areas to different stumping
Source: PeerJ. 2026 May 4;14:e21234. doi: 10.7717/peerj.21234 (PMC13151931; doi:10.7717/peerj.21234)
Supplement: Supplemental Information 2 [file peerj-14-21234-s002.z01 › Data/DATA (3)/richness/avs/Data180.pdf]

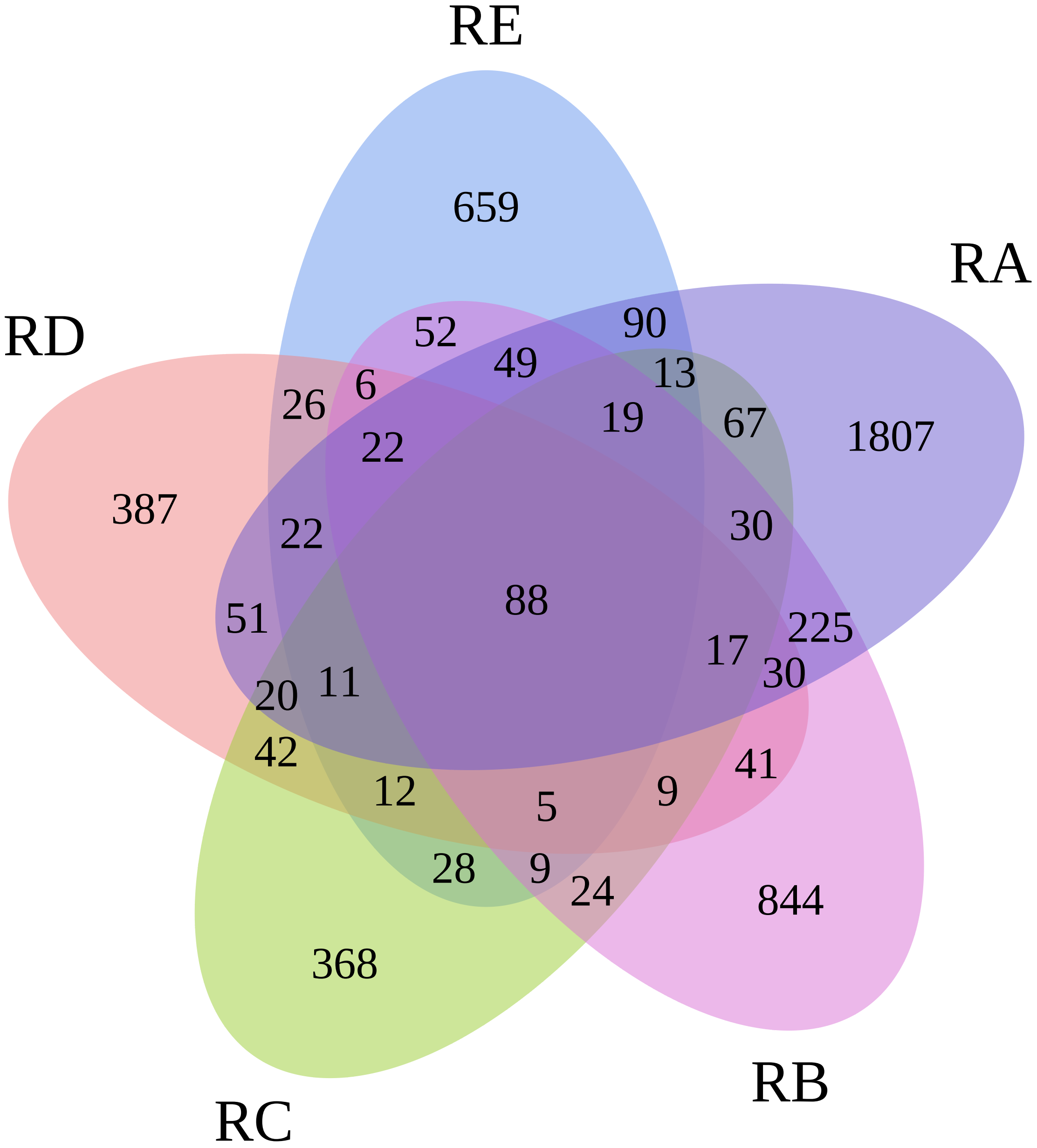
RE
RA
RD
RB
RC
659
52
90
49
13
6
26
19
67
1807
22
387
22
30
88
51
225
17
30
11
20
42
41
12
9
5
28
9
24
844
368
