# Supplementary material for: Response of soil bacterial community structure in Pisha sandstone areas to different stumping
Source: PeerJ. 2026 May 4;14:e21234. doi: 10.7717/peerj.21234 (PMC13151931; doi:10.7717/peerj.21234)
Supplement: Supplemental Information 2 [file peerj-14-21234-s002.z01 › Data/DATA (3)/richness/avs/Data181.pdf]

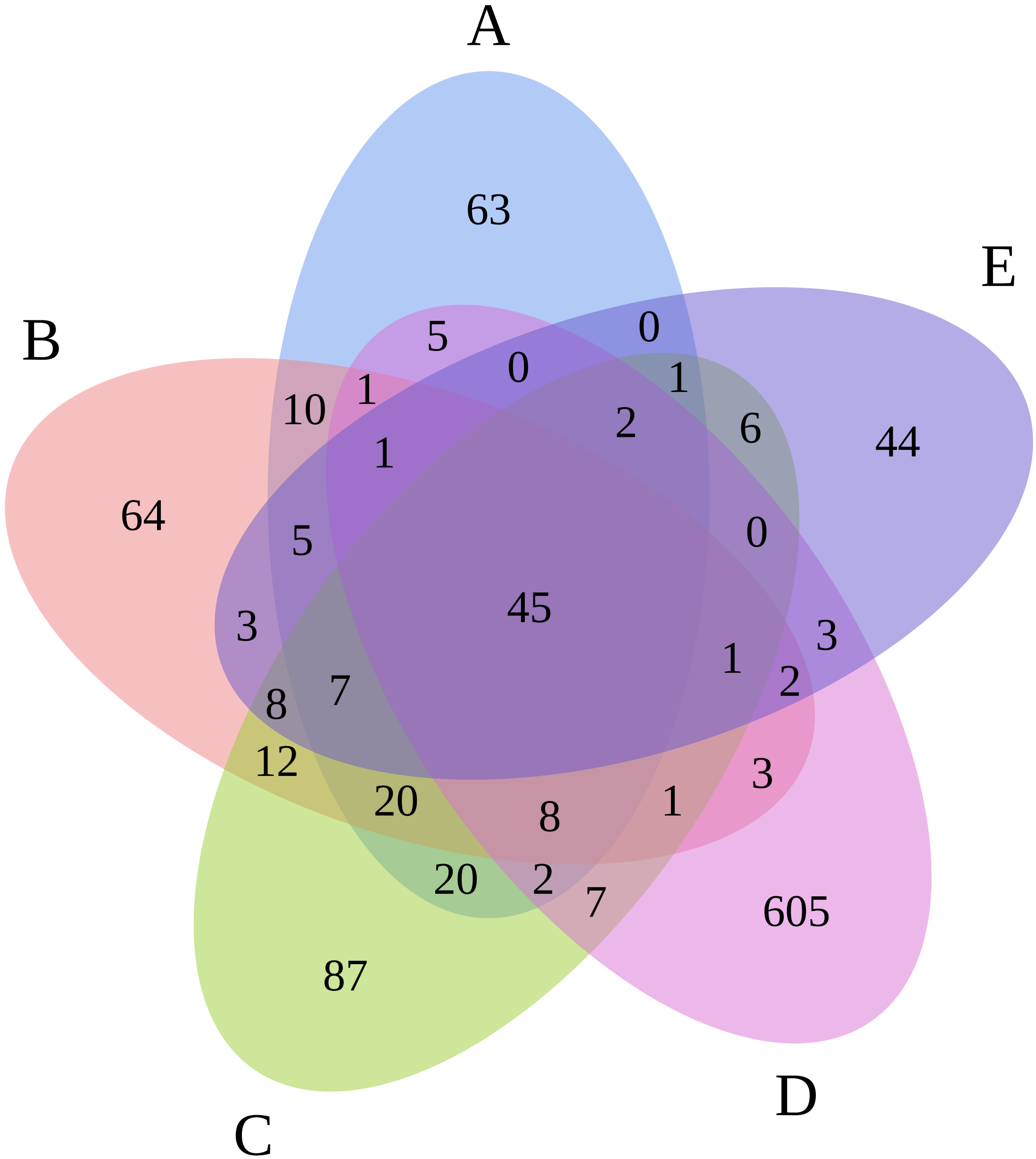
A
B
C
D
E
63
64
87
605
44
5
0
1
10
1
2
6
1
5
0
45
3
1
3
7
2
8
12
3
20
8
1
20
2
7
0
1
