# Supplementary material for: Response of soil bacterial community structure in Pisha sandstone areas to different stumping
Source: PeerJ. 2026 May 4;14:e21234. doi: 10.7717/peerj.21234 (PMC13151931; doi:10.7717/peerj.21234)
Supplement: Supplemental Information 2 [file peerj-14-21234-s002.z01 › Data/DATA (3)/richness/avs/Data184.pdf]

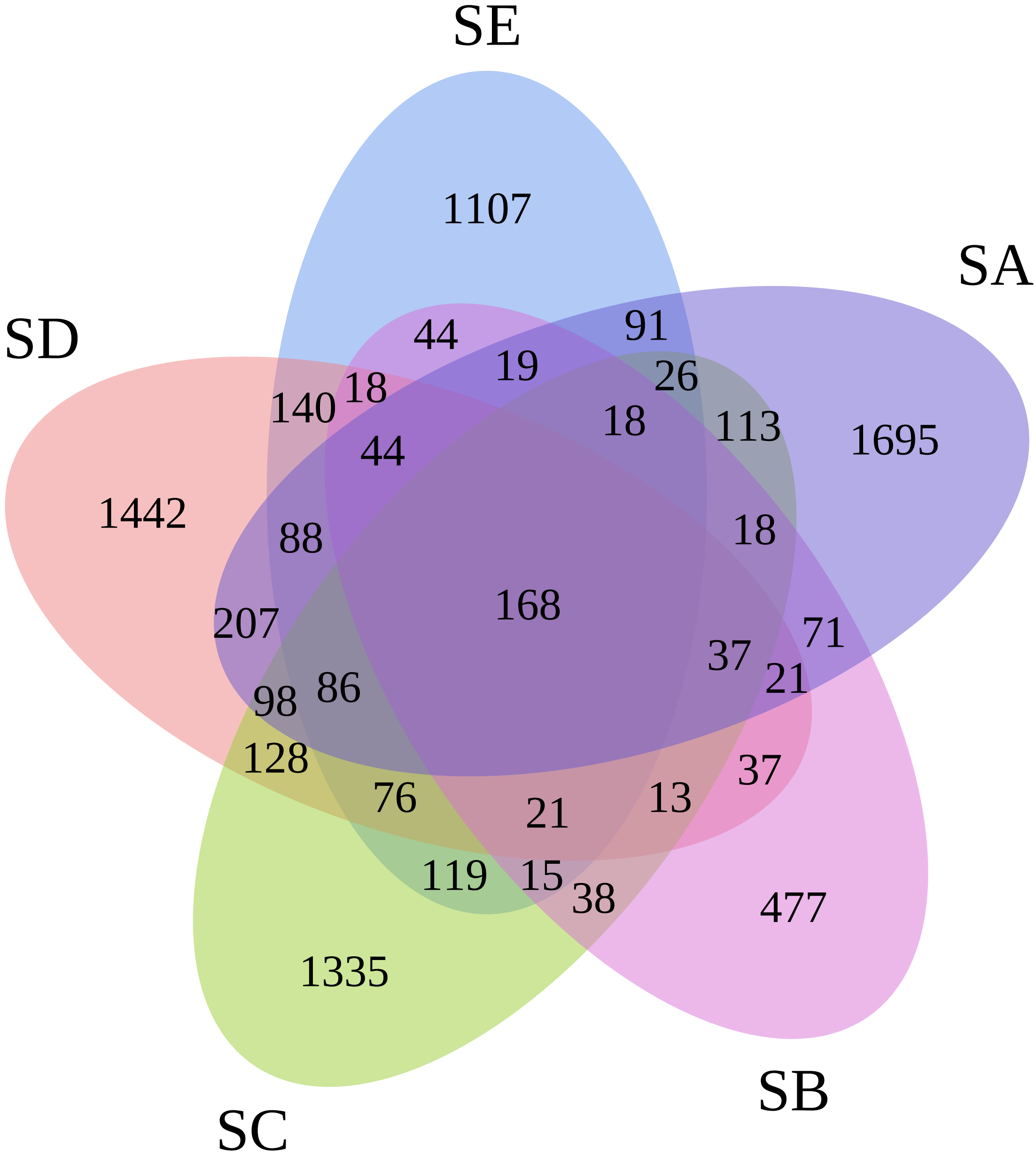
SE
SA
SD
SC
SB
1107
1695
1442
1335
477
44
91
19
26
18
140
18
113
44
88
18
168
207
71
37
86
21
98
128
37
76
13
21
119
15
38
