# Supplementary material for: Response of soil bacterial community structure in Pisha sandstone areas to different stumping
Source: PeerJ. 2026 May 4;14:e21234. doi: 10.7717/peerj.21234 (PMC13151931; doi:10.7717/peerj.21234)
Supplement: Supplemental Information 2 [file peerj-14-21234-s002.z01 › Data/DATA (3)/richness/avs/Data186.pdf]

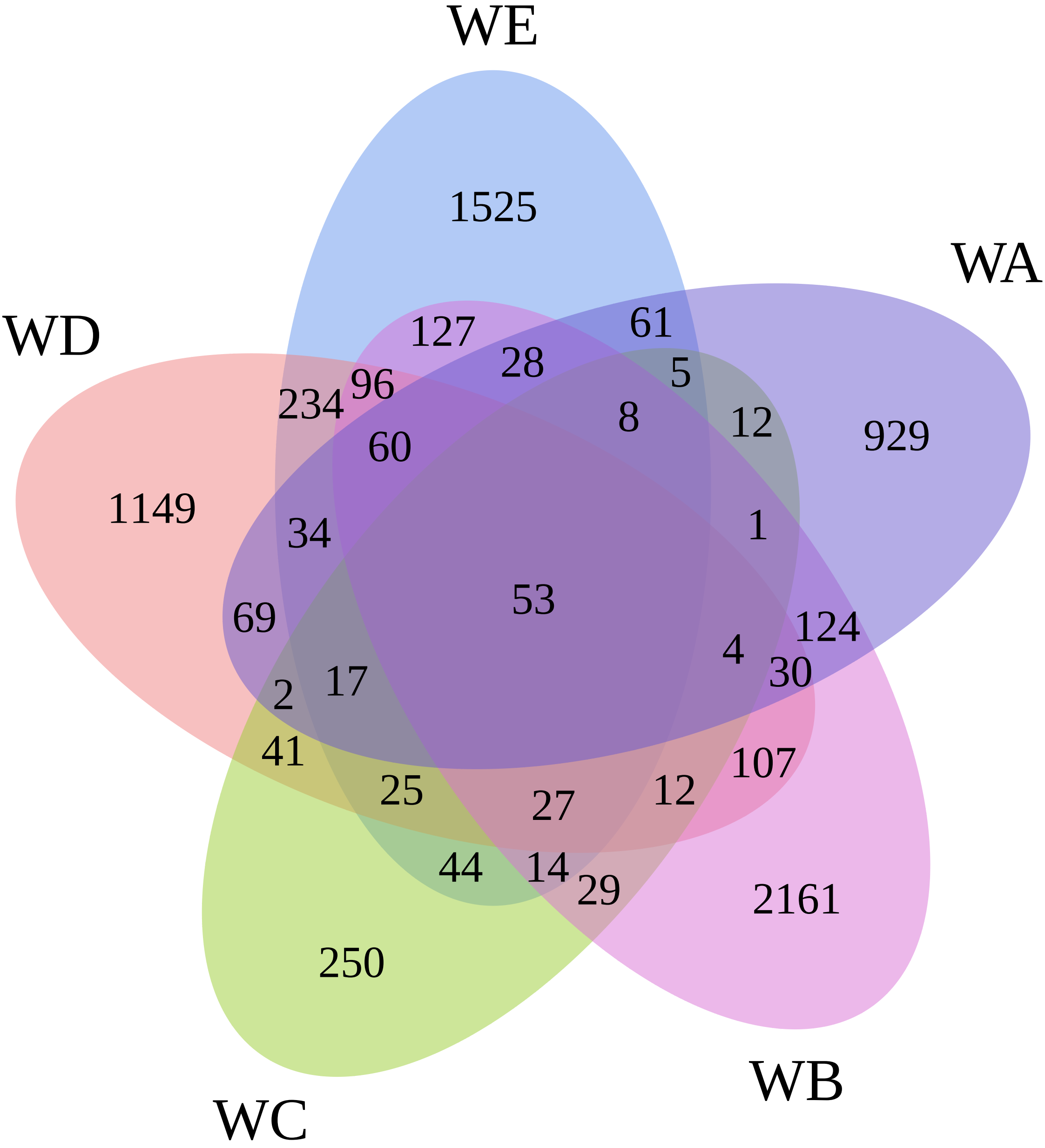
WE
1525
WA
929
WD
1149
WC
250
WB
2161
127
61
28
5
96
234
8
12
60
34
1
53
69
124
4
30
17
2
41
107
25
27
12
44
14
29
