# Supplementary material for: Response of soil bacterial community structure in Pisha sandstone areas to different stumping
Source: PeerJ. 2026 May 4;14:e21234. doi: 10.7717/peerj.21234 (PMC13151931; doi:10.7717/peerj.21234)
Supplement: Supplemental Information 2 [file peerj-14-21234-s002.z01 › Data/DATA (3)/richness/Data176.pdf]

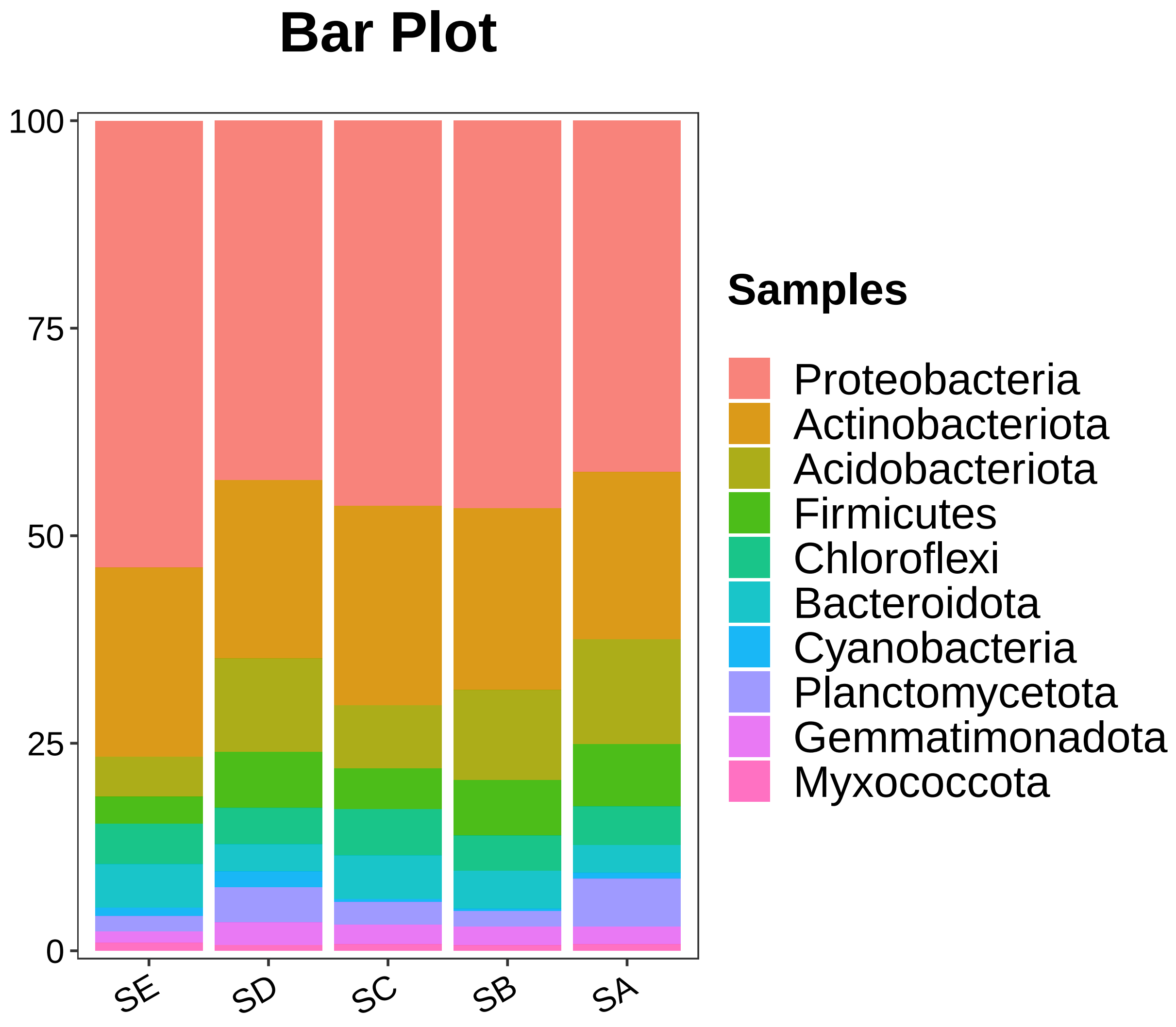
Bar Plot
100
75
50
25
0
SE
SD
SC
SB
SA
Samples
Proteobacteria
Actinobacteriota
Acidobacteriota
Firmicutes
Chloroflexi
Bacteroidota
Cyanobacteria
Planctomycetota
Gemmatimonadota
Myxococcota
