# Supplementary material for: Response of soil bacterial community structure in Pisha sandstone areas to different stumping
Source: PeerJ. 2026 May 4;14:e21234. doi: 10.7717/peerj.21234 (PMC13151931; doi:10.7717/peerj.21234)
Supplement: Supplemental Information 2 [file peerj-14-21234-s002.z01 › Data/DATA/Summary/alpha_diversity/1_rarefaction_curves/All/Data003.pdf]

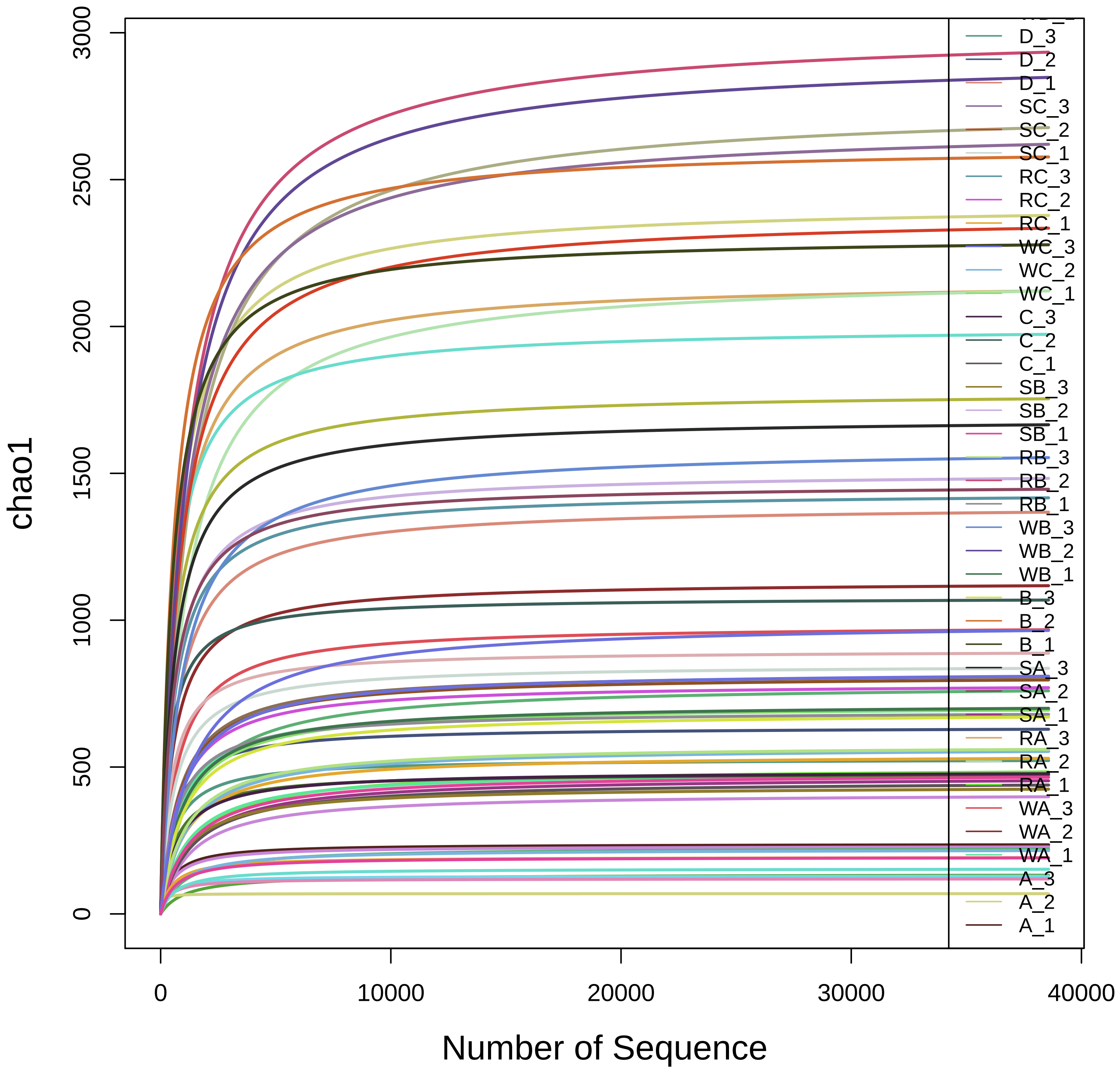

chao1
Number of Sequence
0
500
1000
1500
2000
2500
3000
0
10000
20000
30000
40000
D_3
D_2
D_1
SC_3
SC_2
SC_1
RC_3
RC_2
RC_1
WC_3
WC_2
WC_1
C_3
C_2
C_1
SB_3
SB_2
SB_1
RB_3
RB_2
RB_1
WB_3
WB_2
WB_1
B_3
B_2
B_1
SA_3
SA_2
SA_1
RA_3
RA_2
RA_1
WA_3
WA_2
WA_1
A_3
A_2
A_1
