# Supplementary material for: Response of soil bacterial community structure in Pisha sandstone areas to different stumping
Source: PeerJ. 2026 May 4;14:e21234. doi: 10.7717/peerj.21234 (PMC13151931; doi:10.7717/peerj.21234)
Supplement: Supplemental Information 2 [file peerj-14-21234-s002.z01 › Data/DATA/Summary/alpha_diversity/1_rarefaction_curves/Group/Data014.pdf]

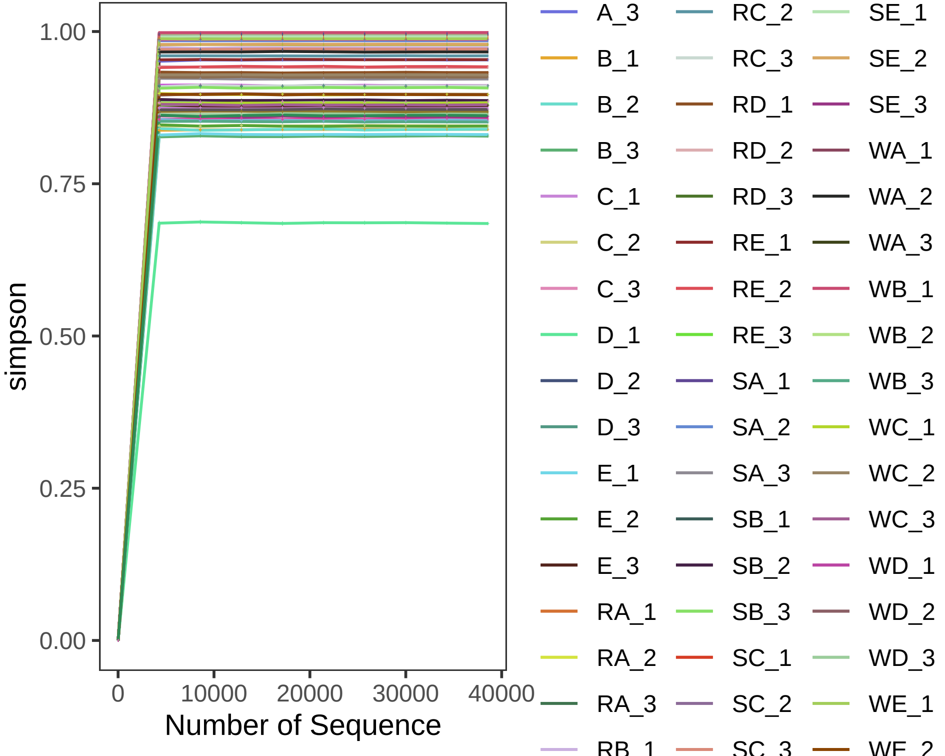

simpson
1.00
0.75
0.50
0.25
0.00
0
10000
20000
30000
40000
Number of Sequence
A_3
B_1
B_2
B_3
C_1
C_2
C_3
D_1
D_2
D_3
E_1
E_2
E_3
RA_1
RA_2
RA_3
RB_1
RC_2
RC_3
RD_1
RD_2
RD_3
RE_1
RE_2
RE_3
SA_1
SA_2
SA_3
SB_1
SB_2
SB_3
SC_1
SC_2
SC_3
SE_1
SE_2
SE_3
WA_1
WA_2
WA_3
WB_1
WB_2
WB_3
WC_1
WC_2
WC_3
WD_1
WD_2
WD_3
WE_1
WE_2
